# A Near-Telomere-to-Telomere Genome Assembly of the Spotted Seal (*Phoca largha*) Reveals Genomic Architecture Underlying Skin and Fur Adaptation

**DOI:** 10.3390/ijms27062618

**Published:** 2026-03-13

**Authors:** Min Zhou, Tingting Li, Xiaotong Zhu, Shenghao Liu, Bailin Cong, Linlin Zhao

**Affiliations:** 1 School of Advanced Manufacturing, Fuzhou University, Jinjiang 362200, China; 18370719071@163.com (M.Z.);; 2 Marine Ecology Research Centre, First Institute of Oceanography, Ministry of Natural Resources, Qingdao 266061, China; ytlitingting@163.com (T.L.);

**Keywords:** near-T2T genome assembly, comparative genome, skin and fur adaptation

## Abstract

The spotted seal (*Phoca largha*) is an ice-associated pinniped in the Northwest Pacific and is a subject of conservation concern under increasing environmental and anthropogenic pressures; however, genomic studies have been constrained by the absence of a high-quality reference genome. Here, we present a near-telomere-to-telomere (near-T2T), gap-free genome assembly of *P. largha* spanning 2.39 Gb and comprising 16 chromosome-length sequences, with a scaffold N50 of 184.39 Mb and high completeness (99.34% complete BUSCOs). Compared with the previous chromosome-level assembly, the new genome improves contiguity and gene-space completeness. Comparative analyses across 20 carnivoran species resolve *P. largha* as sister to *Phoca vitulina* with an estimated divergence time of ~2.1 Ma. Branch-site positive-selection analyses and gene-family evolution analyses identify lineage-associated changes, and enrichment results motivate focused investigation of integument-related gene families. Targeted analyses of keratin (*KRT*) and matrix metalloproteinase (*MMP*) families reveal contrasting chromosomal organisation and evolutionary dynamics: *KRT*s form large chromosomal clusters with broadly conserved synteny across Carnivora but lineage-dependent remodelling within clusters, whereas *MMP*s are dispersed and display largely conserved orthologous correspondence. This high-quality genome provides a high-quality resource for pinniped comparative genomics and for elucidating the genomic architecture of skin and fur adaptation.

## 1. Introduction

The spotted seal (*Phoca largha*, Taxonomy ID: 39090) is an ice-associated pinniped distributed in the Northwest Pacific and occurs in China primarily in the Bohai Sea and the Yellow Sea [1,2]. Because breeding and pup rearing rely on seasonal sea ice, this species is particularly sensitive to climate-driven sea ice loss and increasing anthropogenic disturbance, making it a conservation-relevant sentinel for environmental change in marine ecosystems [3,4]. As a mid–upper-trophic-level predator, *P. largha* also contributes to food web dynamics and ecosystem function in coastal waters [5].

Beyond its ecological significance, *P. largha* and other pinnipeds provide an informative system for dissecting the genetic basis of adaptation to cold and aquatic environments [6,7]. Such adaptation involves coordinated morphological and physiological traits, among which the integumentary system (skin and fur) is central to insulation, barrier integrity, and protection from mechanical and microbial challenges [8,9,10]. Variation in fur properties and skin physiology is therefore directly relevant to thermal balance and habitat use in seasonally ice-covered environments, and understanding its genomic basis can inform both evolutionary and conservation-oriented studies.

High-quality reference genomes with comprehensive annotation are essential for resolving the organisation, regulation, and evolution of trait-relevant loci, particularly in repeat-rich and structurally complex regions that often harbour regulatory elements, segmental duplications, and rapidly evolving gene families [11,12,13,14,15,16]. Although a chromosome-level assembly of *P. largha* has been reported (2.41 Gb, scaffold N50 = 179.7 Mb; accession GWHBJYR00000000), further improvement in structural continuity and telomeric completeness remains desirable [11]. In general, assemblies with incomplete telomeric or repeat resolution may provide limited representation of repeat-dense regions (e.g., centromeres and telomeres) and can complicate detailed structural characterisation. Among closely related phocids, the harbour seal (*Phoca vitulina*; GCF_004348235.1) is one of the most widely accessible references, yet it remains scaffold-level, underscoring the need for a highly contiguous chromosome-scale assembly within this lineage [17].

Recent advances in ultra-long-read sequencing and assembly algorithms have enabled telomere-to-telomere (T2T) genome assemblies that deliver gap-free chromosomes and substantially improve resolution of repetitive DNA and long-range genome architecture [12,16,18,19]. These assemblies provide an enhanced foundation for comparative genomics, including robust inference of gene family organisation, structural evolution, and regulatory landscapes relevant to environmental adaptation [20,21]. In this study, we report a near-T2T genome assembly for *P. largha* using multi-platform sequencing data. The resulting assembly and annotation are further leveraged for comparative genomics, phylogenomics, and gene family analyses across pinnipeds and terrestrial carnivores, with emphasis on integumentary gene families relevant to fur and skin biology. This resource establishes a chromosome-complete genomic framework for future studies of pinniped evolution, cold adaptation, and conservation under ongoing environmental change.

## 2. Results and Discussion

### 2.1. Genome Sequencing and T2T Assembly

A total of 266.78 Gb of DNBSEQ short reads were generated for genome survey analysis and subsequent quality evaluation of the assembly (Table S1a). Based on 17-mer analysis, the genome size was estimated at 2.43 Gb, with heterozygosity of 0.30% and repeat content of 56.86%, consistent with a large and repeat-rich genome (Figure S1a). To generate a highly contiguous assembly and enable chromosome-scale scaffolding, 98.71 Gb of PacBio HiFi, 112.05 Gb of ultra-long ONT, and 262.86 Gb of Hi-C sequencing data were produced (Table S1a).

**Figure 1:**
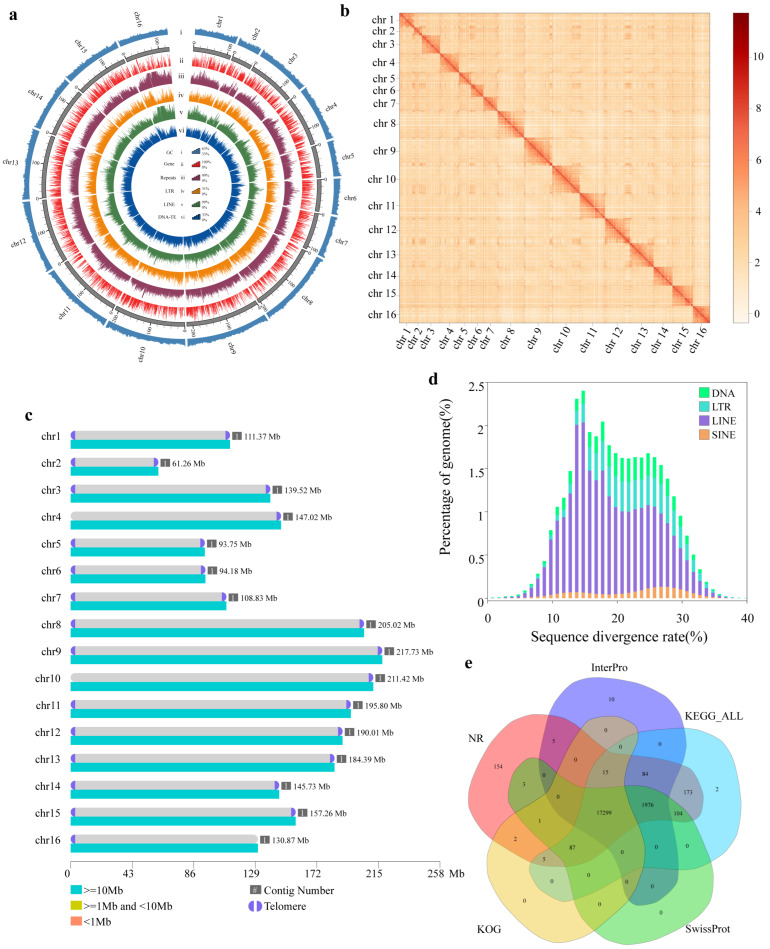
Overview of the *P. largha* genome assembly and annotation. (**a**) Genome features of *P. largha* calculated in non-overlapping 500 kb windows and visualised in a Circos plot (outer to inner tracks): (i) GC content (light blue), (ii) gene density (red), (iii) repeat density (dark brown), (iv) long terminal repeat (LTR) density (dark yellow), (v) long interspersed nuclear element (LINE) density (green), and (vi) DNA transposon density (dark blue). The height of each track represents the corresponding density or content, with taller bars indicating higher values. (**b**) Whole-genome Hi-C contact map at 1 Mb resolution, with interaction strength ranging from weak (light orange) to strong (dark red); darker colours indicate stronger interaction frequencies. (**c**) Chromosomal distribution of contigs with annotated telomere positions in *P. largha*. The different colours of the bars represent contig lengths: light blue indicates contigs greater than 10 Mb; dark yellow indicates contigs between 1 and 10 Mb; light orange indicates contigs less than 1 Mb. The grey bars represent the relative lengths of contigs, ranging from 61.26 Mb to 217.23 Mb. The light purple semi-circular structures represent the telomere structures detected at the ends of the contigs, with the numbers in the grey boxes next to the telomeres indicating the number of contigs in each chromosome, and the adjacent text showing the specific length of each chromosome. (**d**) Divergence landscape of four classes of transposable elements (TEs) annotated by RepeatMasker. The x-axis indicates sequence divergence between annotated TEs and the corresponding consensus sequences in the de novo repeat library, and the y-axis indicates the proportion of genomic TE sequences at each divergence level. The light green represents DNA transposons, light blue represents LTRs, light purple represents LINEs, and light orange represents SINEs. (**e**) Venn diagram summarising functional annotation of predicted genes based on five databases. Different colours represent different datasets: purple represents InterPro, light pink represents NR, light yellow represents KOG, light green represents SwissProt, light blue represents KEGG. The overlapping regions of the petals represent the shared annotations between or among the databases.

The HiFi-based de novo assembly, yielded a 2.40 Gb draft comprising 69 contigs (contig N50 = 84.60 Mb, contig N90 = 29.46 Mb). Hi-C data were then used to scaffold these contigs into chromosome-scale sequences (scaffold N50 = 157.21 Mb), with 99.17% of the assembled sequence anchored. After scaffolding, 572 unplaced sequences remained (total length = 20.10 Mb). These unanchored fragments were further resolved and incorporated using ultra-long ONT reads for contig extension and gap filling, ultimately producing a gap-free assembly of 2.39 Gb composed of 16 chromosome-length contigs, with both contig N50 and scaffold N50 reaching 184.39 Mb (Figure 1a,c; Table 1). The recovery of 16 chromosome-length sequences is consistent with the reported diploid karyotype of *P. largha* (2n = 32), thereby corroborating previous cytogenetic observations [11,22]. Previous assemblies of *P. largha* exhibited several limitations, including high scaffold fragmentation (up to 249,399 scaffolds), lower scaffold N50 values (59.25–142.10 Mb), incomplete chromosome-level anchoring, and reduced BUSCO completeness (89.0–95.0%) (Table S2) [6,11,23,24]. In addition, repeat content estimates varied substantially among earlier versions, suggesting incomplete or inconsistent repeat resolution. These features indicate that previous assemblies were less contiguous and less complete at both structural and gene-content levels.

### 2.2. Quality Evaluation of the P. largha Genome Assembly

The overall quality and completeness of the *P. largha* assembly were evaluated through multiple complementary metrics (Table 1). Mapping rates of DNBSEQ short reads, PacBio HiFi reads, and ONT ultra-long reads reached 99.99%, 100.00%, and 99.95%, respectively, with genome coverage exceeding 99.50%. Merqury k-mer analysis yielded consensus QV scores of 53.77 (DNBSEQ) and 55.82 (PacBio), supporting high consensus accuracy. BUSCO analysis (v5.7.1; --miniprot -m geno -f) recovered 99.34% complete genes from the carnivora_odb12 dataset. The Hi-C contact map showed strong chromosome-scale interaction signals with well-resolved diagonals across all 16 chromosomes, consistent with accurate scaffolding (Figure 1b). Together with telomeric repeat profiling, these results support a chromosome-level, near gap-free genome assembly with high contiguity, accuracy and completeness.

To evaluate chromosomal completeness, canonical telomeric repeats (TTAGGG/CCCTAA) were surveyed at chromosome termini. Telomeric arrays were detected at 29 of the 32 chromosome ends (4-2907 repeat units), whereas no canonical arrays were identified at the 5′ ends of chr4 and chr10 and the 3′ end of chr16 (0 units) (Table 2). The absence of detectable canonical repeats at these termini may reflect biological variation in telomeric repeat structure or limitations in assembling highly repetitive terminal sequences. Putative centromeric regions were inferred based on the distribution of extensive tandem repeat arrays identified by TRF. A single major centromeric region was detected on each chromosome, with lengths ranging from 146,815 bp (chr12) to 30,065,961 bp (chr8) (Table S3). These regions were enriched in clustered tandem repeat units and were primarily located at internal chromosomal positions, except for chr6, where the repeat array was positioned near the chromosomal terminus.

Importantly, ultra-long ONT reads (maximum length = 724 kb; N50 = 125 kb for reads ≥ 100 kb) provided approximately 10× coverage of the genome and supported sequence continuity across highly repetitive regions, including telomeric and centromeric repeat arrays (Table S1b). Collectively, these results indicate that the assembly approaches a telomere-to-telomere configuration for most chromosomes and represents a high-quality, near-T2T assembly supported by ultra-long read continuity across repetitive regions.

### 2.3. Gene Annotation

The *P. largha* genome contained approximately 955.49 Mb of repetitive sequences, accounting for 39.91% of the total assembly (Figure 1d; Table S4). Among these elements, long interspersed nuclear elements (LINEs) were the most abundant (27.76%), followed by long terminal repeats (LTRs, 5.79%) and DNA transposons (3.84%). In addition, the analysis identified a total of 1,611,509 SSR loci in the *P. largha* genome, corresponding to a density of approximately 673 SSRs per Mb. Among these, mononucleotide repeats were the most abundant, accounting for 58.97% of all SSRs, followed by dinucleotide repeats (31.27%). Trinucleotide, tetranucleotide, pentanucleotide, and hexanucleotide repeats comprised 3.36%, 5.53%, 0.68%, and 0.21%, respectively. In addition, 280,887 SSRs (17.43%) were identified as compound microsatellites (Figure S2; Table S5).

Gene prediction and functional annotation were performed on the repeat-masked assembly, yielding 20,037 high-confidence protein-coding genes, of which 99.42% were functionally annotated through homology-and domain-based searches (Figure 1e). The predicted gene models in *P. largha* assembly showed similar distribution patterns in gene length, coding sequence length, exon length, and intron length compared to other five closely related species (Figure S1b), with no obvious shift in gene structure. In addition, 42,205 non-coding RNA (ncRNA) genes were identified, including 821 microRNAs (miRNAs), 698 transfer RNAs (tRNAs), 416 ribosomal RNAs (rRNAs), and 2638 small nuclear RNAs (snRNAs) (Table S6). Among these, snRNAs constituted the most abundant class.

### 2.4. Genomic Comparisons of P. largha with Other Carnivorans

**Figure 2:**
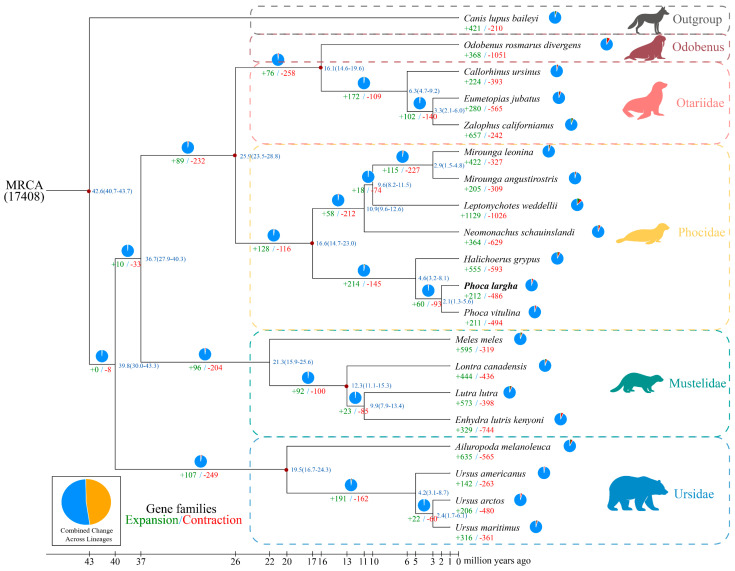
Comparative genomic analysis of *P. largha* and related carnivorans. A maximum-likelihood phylogeny was inferred from single-copy orthologues across 20 species. Numbers along branches indicate the numbers of expanded (green) and contracted (red) gene families. The inset pie chart summarises overall expansion and contraction of gene families, with the yellow sector representing families showing expansion or contraction in at least one lineage or ancestral node and the blue sector representing families conserved across all lineages. Divergence times (blue numbers, Ma) are shown at internal nodes, with values in parentheses indicating 95% highest posterior density (HPD) intervals. Red dots denote fossil calibration points used for time calibration.

Genomic comparisons were conducted across 20 carnivoran genomes, including 11 pinnipeds, 8 terrestrial carnivores, and *C. lupus baileyi* as the outgroup (Table S7). OrthoFinder clustering identified 13,256 orthogroups shared among all species, from which 9130 single-copy orthologues were selected for phylogenetic analyses (Figure S1c). In total, 98.61% (19,758 genes) of predicted genes in *P. largha* were assigned to 17,967 gene families, with an average of 1.1 genes per family, indicating a highly conserved gene repertoire typical of mammalian core genomes. Only 33 genes, distributed across nine gene families, were species-specific, suggesting limited lineage-specific gene emergence. The resulting maximum-likelihood topology was broadly congruent with previous reconstructions based on nuclear and mitochondrial data, providing genome-wide support for the inferred relationships [25]. *P. largha* was resolved as sister to *P. vitulina*, with an estimated divergence time of ~2.1 Ma (Figure 2). Molecular dating placed the divergence between *P. largha* and *P. vitulina* in the late Pliocene to early Pleistocene (~2.1 Ma), a period characterised by pronounced climatic oscillations and glacial expansion in the Northern Hemisphere. Although direct causal relationships cannot be inferred, this temporal framework is broadly consistent with scenarios in which climatic and geographic changes contributed to lineage diversification within Phocidae [26,27].

Genome-wide synteny analyses were performed between *P. largha* and two representative pinnipeds (*Z. californianus* and *N. schauinslandi*) to evaluate chromosomal conservation and large-scale structural evolution (Figure S1d; Table S8). A total of 394 syntenic blocks were detected in the *P. largha* self-comparison, whereas 325 and 301 blocks were recovered in the interspecific comparisons, respectively. Interspecific blocks contained more syntenic gene pairs per block (mean = 61–65) than blocks detected in the self-comparison (mean = 8), consistent with the recovery of large collinear segments in interspecies comparisons. Median block lengths further supported broadly conserved macrosynteny, while also indicating some degree of lineage-specific reorganisation. Collectively, these results indicate that the chromosomal architecture of *P. largha* is largely conserved relative to other pinnipeds, with structural divergence concentrated in a subset of genomic regions. Notably, the limited number of species-specific genes and the predominance of conserved gene families suggest that lineage-specific evolutionary changes in *P. largha* are more likely to involve copy number variation and sequence-level modification within existing gene families, rather than extensive de novo gene emergence.

Expansion and contraction of gene families were inferred in this study. On the *P. largha* lineage, 212 expanded and 486 contracted gene families were identified, among which 149 families (612 genes) and 228 families (247 genes) were significantly expanded and contracted, respectively (*p* < 0.05) (Figure 2). GO enrichment of genes from significantly expanded families highlighted terms related to integumentary and extracellular matrix processes, including keratinisation (GO: 0031424), keratin filament (GO: 0045095), intermediate filament organisation (GO: 0045109) and extracellular matrix organisation (GO: 0030198) (Table S9). KEGG enrichment further indicated that these expanded-family genes were over-represented in pathways relevant to epithelial integrity and tissue remodelling, including Tight junction (ko04530), Cell adhesion molecules (ko04514), PI3K-Akt signalling pathway (ko04151) and Wnt signalling pathway (ko04310) (Table S10). Together, these enrichments are consistent with lineage-specific copy number changes involving gene sets associated with epithelial barrier function and extracellular organisation.

In parallel, 474 positively selected genes (PSGs) were identified on the *P. largha* lineage (Table S11). GO enrichment of PSGs included terms related to epidermal structure and associated functions (e.g., GO: 0031424 and GO: 0045095) (Table S12). In addition, KEGG enrichment of PSGs recovered pathways related to epithelial organisation and signalling (e.g., ko04530, ko04514 and ko04151) (Table S13). Collectively, these results suggest coordinated evolutionary change at both the copy number turnover level (gene-family expansion/contraction) and the sequence level (positive selection), potentially involving gene sets associated with integumentary structure and epithelial function in *P. largha*. The recurrence of similar enrichment patterns across analyses may indicate multi-level evolutionary modulation within integumentary-associated gene networks.

Given the repeated enrichment of skin- and fur-related functions, subsequent analyses focused on two representative gene families with established roles in integumentary structure and remodelling, keratins (*KRT*s) and matrix metalloproteinases (*MMP*s), to characterise their evolutionary dynamics, genomic organisation, and lineage-specific features in *P. largha*. Within the keratin family, variation in gene length and predicted physicochemical properties was observed, suggesting functional diversification among paralogues. This diversity may contribute to structural heterogeneity within keratinised tissues, although direct functional consequences remain to be established.

In contrast, the *MMP* gene family exhibited relatively conserved gene numbers and structural features, consistent with functional constraint on extracellular matrix remodelling enzymes. Positive selection signals within this family may therefore reflect fine-scale adjustment of enzymatic or regulatory properties rather than large-scale gene expansion.

### 2.5. Analyses of KRT and MMP Gene Families

Across the 20 carnivoran genomes, 916 *KRT* genes and 406 *MMP* genes were identified. In *P. largha*, 48 *KRT*s were recovered (33 type I and 15 type II), whereas the *MMP* repertoire comprised 20 members and was strongly enriched for WithHemopexin members (18/20), with only two WithoutHemopexin genes detected (Table S14). The marked excess of *KRT*s over *MMP*s is consistent with the large, structurally partitioned nature of the keratin family relative to the typically smaller, functionally specialised *MMP* family.

**Figure 3:**
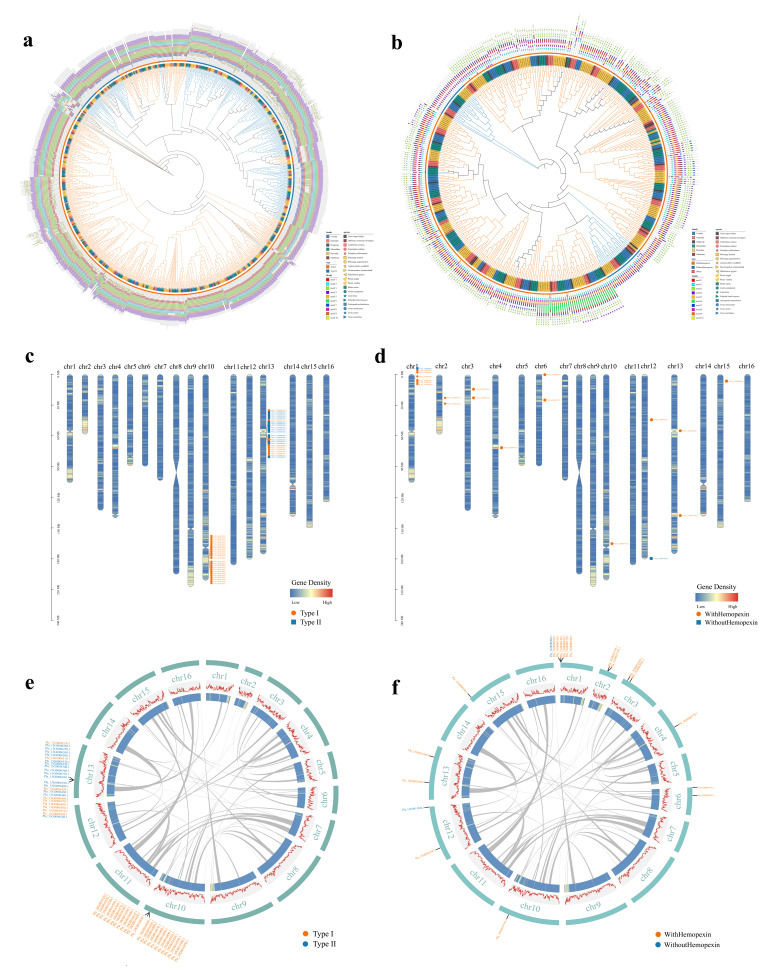
Overview of the *P. largha* gene family analysis results. (**a**,**b**) Phylogenetic analysis of the *KRT* and *MMP* gene families. The outermost circle shows motif analysis, with different colours representing distinct motifs identified in the *KRT* and *MMP* families. The arrangement of motifs indicates their relative positions. The inner circle represents the evolutionary relationships of *KRT* and *MMP* genes among *P. largha* and 19 other species, with branch colours and stripe colours representing gene family types. In (**a**), orange represents *KRT* type I and blue represents *KRT* type II; in (**b**), orange represents *MMP*, blue represents *MMP* with Hemopexin, and light red represents Others. The innermost circle represents species, with leaf colours on the phylogeny representing different families: blue for Ursidae, light red for Otariidae, grey for the outgroup, dark green for Mustelidae, dark yellow for Phocidae, and brown for Odobenidae. With the symbol in front of the number denoting the species, the numbers on the leaves represent specific gene IDs within particular species. (**c**,**d**) Distributions of *KRT*s and *MMP*s on the *P. largha* chromosomes chr01–chr16. The left scale represents chromosome length, and the heatmaps represent gene density on the chromosomes, with gene density calculated for each 500 kb window. Gene density is represented by a gradient from low (blue) to high (red). The constrictions represent the relative positions of centromeres. In (**c**), orange circles and blue squares represent the relative positions of *KRT* type I and *KRT* type II, respectively. In (**d**), orange circles and blue squares represent the relative positions of *MMP* with Hemopexin and *MMP* without Hemopexin, respectively. (**e**,**f**) Collinearity analysis of *KRT*s and *MMP*s within *P. largha*. The outermost circle in the Circos plot represents chromosome relative length and the locations of *KRT* and *MMP* genes. The second circle shows GC content as a line graph, with higher values indicating higher GC content. The heatmaps represent the gene density distribution, with gene density calculated for each 500 kb window, and the grey connecting lines indicate the syntenic blocks of *P. largha*.

Phylogenetic inference indicated that *P. largha KRT*s separated largely into the canonical type I and type II groups and were interspersed with orthologues from other carnivores (Figure 3a), supporting conservation of the main *KRT* lineages across Carnivora. Motif patterns were broadly similar within major clades, consistent with shared requirements for intermediate-filament assembly, but clade-specific differences in motif presence and arrangement were apparent, suggesting diversification concentrated in the variable head/tail regions rather than the filament-forming backbone. Notably, motif 1 showed clade-dependent variation in copy number and position; only N-terminal instances coincided with the Keratin_2_head region (Figure 3a; Figure S3), whereas C-terminal occurrences lay outside this domain, indicating positional heterogeneity in motif usage. Several type I *KRT* lineages formed distinct clades lacking motif 7, whereas mixed clades containing both type I and type II members, together with several minor subclades, typically retained motif 7 (Figure 3a), consistent with subfamily-specific constraint alongside lineage-dependent turnover of non-core elements.

For the *MMP* family, WithHemopexin genes predominated in *P. largha* and clustered with homologues from other carnivores (Figure 3b), indicating that major *MMP* subfamily relationships are broadly conserved. Motif compositions were likewise coherent within clades, and WithHemopexin and WithoutHemopexin groups were distinguished by characteristic motif combinations. Motifs 3, 6 and 10 were largely restricted to particular WithHemopexin-associated clades, with lineage-specific variation in copy number and relative position (Figure 3b), consistent with a modular architecture in which diversification is more readily accommodated in accessory regions than in the catalytic core.

Domain annotations reinforced these patterns. Most *KRT* proteins contained the conserved Filament (or Filament superfamily) domain, whereas the Keratin_2_head region varied in length and copy number among members; additional domains (e.g., Keratin_B2_2 and Keratin_2_tail superfamily) occurred in a subset of *KRT*s, indicating subfamily-restricted architectures. Consistent with the motif analysis, N-terminal motif 1 corresponded to the Keratin_2_head region (Figure S3). In *MMP*s, most proteins contained the core Peptidase_M10 and HX domains, whereas the PG_binding_1 domain varied in copy number and length. A subset of M10 regions was extended and contained three FN2 domains, consistent with additional matrix-interaction modules, and the ZnMc_*MMP* domain occurred in WithHemopexin members. In agreement with this domain variation, motif 6 corresponded to the PG_binding_1 region (Figure S4), linking clade-specific motif patterns to differences in the substrate-binding portion of the proteins.

Chromosomal localisation revealed contrasting genomic organisation between the two families. *KRT* genes were strongly clustered on chr13 and chr15, with type I *KRT*s enriched towards the distal region of chr13 and the medial region of chr15, whereas type II *KRT*s were largely confined to the medial region of chr15 (Figure 3c). Such clustering is consistent with local organisation of keratin loci and motivates subsequent examination of whether local duplication and rearrangement contributed to lineage-specific variation [28,29]. This clustered arrangement may also facilitate coordinated regulation among neighbouring keratin genes, as observed in other vertebrate keratin loci.

By contrast, *MMP* genes were dispersed across multiple chromosomes (absent only from chr5, chr7, chr8, chr9, chr11, chr14 and chr16), with substantial heterogeneity in chromosomal copy number; chr1 harboured the largest set (six WithHemopexin and one WithoutHemopexin gene), whereas chr4, chr10 and chr15 each contained a single *MMP* gene (Figure 3d). This dispersed distribution is consistent with diversified regulatory contexts and provides a framework for interpreting the intra- and interspecies collinearity patterns described below. Such spatial separation may reflect stronger functional constraint on independent transcriptional control of proteolytic enzymes.

Consistent with the chromosomal organisation of the two families, intraspecies collinearity analysis detected no obvious paralogous collinear blocks involving either *KRT*s or *MMP*s (Figure 3e,f). This pattern argues against recent, large-scale segmental duplication as the primary driver of copy number differences in these families in *P. largha*. Instead, any lineage-specific changes are more plausibly attributable to local-scale processes (e.g., tandem duplication, small deletions, and gene conversion) that are common in multigene families, particularly within repeat-rich genomic neighbourhoods [30,31,32,33]. It should also be noted that the absence of detectable blocks does not exclude small or ancient duplication events that fall below the sensitivity of collinearity-based detection, emphasising the importance of complementary evidence from structural variant and copy number analyses [34].

**Figure 4:**
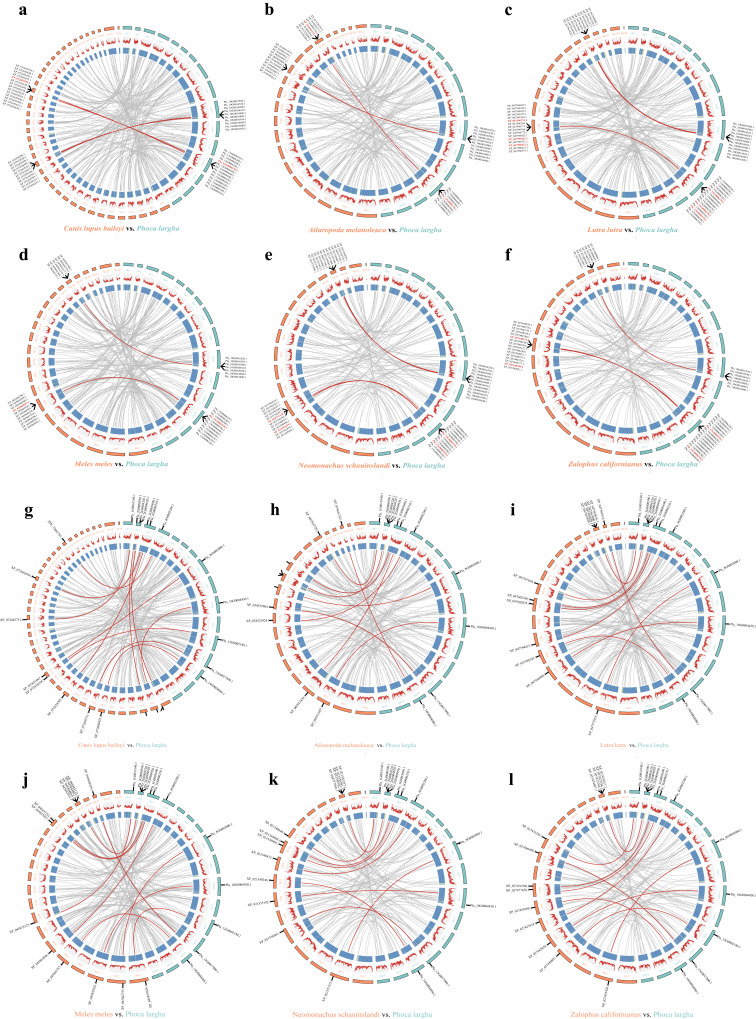
Comparative synteny of the keratin (*KRT*) and matrix metalloproteinase (*MMP*) gene families between *P. largha* and six representative carnivoran species. In each Circos plot, chromosomes of *P. largha* are shown in light blue, and chromosomes of the comparison species are shown in orange. The outermost ring indicates relative chromosome lengths and the positions of *KRT*/*MMP* genes; the second ring shows GC content as a line plot (higher values indicate higher GC content); and the heatmap tracks show gene density calculated in non-overlapping 500 kb windows (low to high). Grey links denote all detected collinear gene pairs between species, whereas dark red links highlight orthologous pairs belonging to the focal gene family (*KRT* in (**a**–**f**); *MMP* in (**g**–**l**)). Panels (**a**–**f**) show syntenic relationships for the *KRT* family, and panels (**g**–**l**) show those for the *MMP* family. Comparison species are: (**a**,**g**) *Canis lupus baileyi*, (**b**,**h**) *Ailuropoda melanoleuca*, (**c**,**i**) *Lutra lutra*, (**d**,**j**) *Meles meles*, (**e**,**k**) *Neomonachus schauinslandi*, and (**f**,**l**) *Zalophus californianus*.

In interspecies comparisons, the *KRT* family exhibited a clear contrast between conserved cluster-level organisation and lineage-dependent disruption of local order. The two major *KRT* clusters in *P. largha*, on chr10 (Pla_10G0016520.1-Pla_10G0016620.1) and chr13 (Pla_13G0006230.1-Pla_13G0006540.1), showed strong syntenic conservation with most comparator species, including *C. lupus baileyi*, *A. melanoleuca*, *M. meles* and *N. schauinslandi* (Figure 4a–e). In these lineages, *KRT* orthologues mapped predominantly to two continuous blocks, with mainly one-to-one or few-to-one correspondences to *P. largha* loci, indicating that the overall cluster architecture and much of the underlying gene content have been retained. Such preservation suggests that the broad genomic framework of keratin clusters is evolutionarily stable across diverse carnivoran lineages. By contrast, *L. lutra* and *Z. californianus* displayed more fragmented patterns (Figure 4c,f), with *KRT* orthologues distributed across multiple discontinuous segments and showing frequent one-to-many correspondences and disrupted local order within both clusters. This contrast indicates that, while the macro-organisation of keratin loci may be broadly conserved, fine-scale gene order within clusters can be comparatively labile in certain lineages. Two non-mutually exclusive interpretations are supported by these signatures: (i) biological reorganisation, where recurrent unequal recombination and local rearrangements within *KRT* arrays alter gene order and generate lineage-specific expansions/retentions; and/or (ii) technical fragmentation, where assembly and annotation differences disproportionately affect keratin clusters because they are enriched for highly similar paralogues and flanking repeats. The predominance of one-to-many correspondences is particularly compatible with either recent lineage-specific duplication/retention in the comparator genome(s) or collapsed/expanded representations of highly similar loci, and therefore warrants careful interpretation alongside assembly continuity and gene-model curation.

Taken together, these interspecific comparisons suggest that keratin gene clusters combine long-term positional conservation with lineage-dependent flexibility at the level of local arrangement and copy number. This dual pattern is consistent with the view that clustered structural-protein families may retain stable genomic compartments while accommodating gradual internal turnover.

In contrast to the cluster-based organisation of keratins, interspecies collinearity for the *MMP* family largely reflected its dispersed genomic distribution. Orthologous *MMP* loci in *P. largha* were detected across multiple chromosomes (chr1, chr2, chr3, chr4, chr6, chr10, chr12, chr13 and chr15), mirroring the multi-chromosomal organisation characteristic of this family. Across all six comparators, most *MMP* orthologues exhibited clear one-to-one or few-to-one correspondences to *P. largha* loci (Figure 4g–l), indicating that gene content and broad chromosomal placement are comparatively conserved despite divergence among carnivoran lineages.

This pattern suggests that evolutionary constraint within the *MMP* family may operate primarily at the level of individual gene identity and catalytic function, rather than through maintenance of specific chromosomal neighbourhoods. Such stability is consistent with functional constraint on extracellular proteases, for which dosage imbalance and pleiotropic effects may limit extensive copy number volatility.

Several orthologous relationships were consistently preserved across all species, particularly those anchored to genomic regions corresponding to *P. largha* chr1 (Pla_1G0000090.1, Pla_1G0000110.1), chr2 (Pla_2G0002730.1, Pla_2G0003150.1), chr3 (Pla_3G0001650.1, Pla_3G0002920.1), chr4 (Pla_4G0006770.1), chr6 (Pla_6G0000260.1, Pla_6G0003570.1), chr12 (Pla_12G0002740.1, Pla_12G0015830.1), chr13 (Pla_13G0005260.1, Pla_13G0014340.1) and chr15 (Pla_15G0000560.1). The recurrence of these conserved loci across multiple species further supports the relative positional stability of core *MMP* genes.

Taken together, the contrasted collinearity signatures of *KRT* and *MMP* families suggest that integument-associated genomic evolution in *P. largha* may involve structurally distinct modes of organisation. Keratin genes appear to be embedded within stable genomic clusters that retain conserved macro-architecture while permitting localised internal rearrangement, whereas *MMP* genes are dispersed and exhibit conservation primarily at the level of individual loci rather than clustered neighbourhoods. These contrasting architectures illustrate how different multigene families can follow distinct structural evolutionary trajectories within the same genome.

## 3. Materials and Methods

### 3.1. Sample Collection and Sequencing

A healthy adult female spotted seal (*Phoca largha*) from Haichang Polar Ocean World (Qingdao, China) was selected for T2T genome assembly. Peripheral blood was collected in K_2_EDTA tubes (KWS, Shijiazhuang, China), stored at 4 °C, and processed within 24 h. High-molecular-weight genomic DNA was extracted using the MagMAX™ DNA Multi-Sample Ultra Kit (Thermo Fisher Scientific, Waltham, MA, USA). DNA purity and concentration were assessed with a NanoDrop spectrophotometer (Thermo Fisher Scientific) and a Qubit 2.0 fluorometer (Thermo Fisher Scientific), and integrity was verified by pulsed-field gel electrophoresis (PFGE).

Four sequencing libraries were constructed: (i) short-insert paired-end libraries (350 bp) sequenced on the DNBSEQ-T7 platform (MGI Tech, Shenzhen, China); (ii) PacBio HiFi libraries (~20 kb; SMRTbell Express Template Prep Kit 2.0) sequenced on the Sequel II system (Pacific Biosciences, Menlo Park, CA, USA); (iii) ultra-long Oxford Nanopore Technologies (ONT) libraries (~200 kb; Ultra-Long DNA Sequencing Kit) sequenced on the PromethION platform (Oxford Nanopore Technologies, Oxford, UK); and (iv) Hi-C libraries sequenced on the DNBSEQ-T7 platform [35].

In parallel, total RNA was extracted from the same blood sample using the GeneJET RNA Purification Kit (Thermo Fisher Scientific). RNA purity, concentration, and integrity were evaluated using a NanoDrop spectrophotometer, a Qubit fluorometer, and an Agilent 2100 Bioanalyser (Agilent Technologies, Santa Clara, CA, USA), respectively. Only RNA samples with RIN ≥ 7 were used for cDNA synthesis and library construction. RNA libraries passing quality control were sequenced on the DNBSEQ-T7 platform to support gene annotation. All library preparation and sequencing were performed by OneMore Tech Ltd. (Wuhan, China).

### 3.2. Genome Assembly and Assessment

Following quality filtering, DNBSEQ short reads were used for k-mer analysis (k = 17) with GCE (v1.0.0; -c 95 -H 1) to estimate genome size, heterozygosity, and repeat content [36]. PacBio subreads were processed using SMRT Link (v11.0.0) to generate circular consensus sequencing (CCS/HiFi) reads [37]. Filtered HiFi reads were assembled using hifiasm (v0.19.9), and redundant haplotigs were removed with purge_haplotigs (v1.1.3; -a 70 -j 80 -d 200) [38,39].

Hi-C reads were processed with HiCUP (v0.7.2; -NM 3) and Juicer (v1.6) to obtain contact maps [40,41]. Chromosome-scale scaffolding was performed using 3D-DNA (v180922; -r 0), followed by manual curation in Juicebox (v1.11.08) [42,43]. Haplotype-aware scaffolding was refined using HapHiC (v1.0.2; -NM 3) [44]. Ultra-long ONT reads were aligned to the draft genome with Minimap2 (v2.24; -ax map-ont), and consensus polishing of ONT-supported regions was carried out using Medaka (v1.7.2; model r941_prom_fast_g507) [45,46]. Telomeric regions were extended where supported by ONT alignments, and remaining gaps were closed using TGS-GapCloser (v1.2.0; --min_nread 10) [47]. Final short-read polishing was performed with Pilon (v1.23; --fix all --changes) after aligning DNBSEQ reads with BWA (v0.7.12-r1039; -M), iterating until Merqury QV and BUSCO scores plateaued [48,49]. This workflow generated a gap-free, chromosome-level assembly comprising 16 chromosomes.

Assembly quality was evaluated through multiple complementary metrics. DNBSEQ short reads were aligned with BWA, and PacBio HiFi and ONT long reads were aligned with Minimap2 to assess mapping rates and coverage. Consensus accuracy was estimated using Merqury (v1.3.1) with 21-mer databases derived from DNBSEQ and PacBio HiFi reads to report the quality value (QV) and k-mer completeness [50]. Gene completeness was assessed with BUSCO (v5.7.1; --miniprot m geno -f) against the Carnivora_odb10 dataset [51]. Telomeric repeats (TTAGGG/CCCTAA) were identified with quartet (v1.1.4); a chromosome end was considered telomeric if more than 5 kb of canonical repeats were present within the terminal 50 kb on either strand. Centromeric regions were also investigated with Quartet by screening for putative centromeric tandem repeat arrays across the genome. Regions containing long arrays of centromere-associated tandem repeats were defined as candidate centromeric regions [52].

### 3.3. Genome Annotation and Prediction

Repetitive elements were annotated by combining homology-based methods and de novo prediction. For homology comparison, RepeatMasker (open-4.0.9; -nolow -no_is -norna) and its built-in RepeatProteinMask (v3.2.2) were used to identify interspersed repeats against the RepBase database (26 October 2018) [53,54]. For de novo prediction, TRF (v4.09; 2 7 7 80 10 50 2000 d -h) was employed to detect the tandem repeats [55]. In addition, a species-specific repetitive sequence library was constructed with RepeatModeler (open-1.0.11) and LTR-FINDER_parallel (v1.0.7) [56,57]. The combined repeat library was subsequently used as input for RepeatMasker to generate a unified repeat annotation. Redundant and overlapping repeat annotations identified by different methods were resolved during the RepeatMasker-based integration, and only non-overlapping genomic regions were retained for further analysis. In addition, Simple sequence repeats (SSRs) were identified using MISA (v2.1) [58]. The minimum numbers of repeat units were defined as 10 for mononucleotide repeats, 6 for dinucleotide repeats, and 5 for tri-, tetra-, penta-, and hexanucleotide repeats, respectively.

Protein-coding genes were predicted by integrating de novo, homology-based, and transcriptome evidence. For de novo prediction, the repeat-masked genome assembly was subjected to gene prediction using AUGUSTUS (v3.2.2), GeneScan (v1.0), and GlimmerHMM (v3.04) [59,60,61]. For homology-based prediction, proteomes from related species (*Phoca vitulina*, *Mirounga angustirostris*, *Neomonachus schauinslandi*, *Panthera leo*, *Canis lupus familiaris*) were downloaded from the NCBI genome database (https://www.ncbi.nlm.nih.gov/datasets/genome, accessed on 1 April 2025) and aligned with Miniprot (v0.11-r234; --gff-only -O 11 -E 1 -F 23 -C 1 -B 5 -G 200,000 -j 1) (Table S7) [62]. Gene annotations were further transferred using Liftoff (v1.6.3; -a 0.5 -s 0.5) to refine exon–intron boundaries [63]. For transcriptome-supported prediction, RNA-seq reads were aligned using HISAT2 (v2.1.0; --dta), transcripts were assembled with StringTie (v1.3.5), and coding sequences were identified with TransDecoder (v5.5.0) [64,65,66]. Assemblies from Cufflinks (v2.2.1; -e 100) were incorporated as supplementary evidence [67]. All gene models were integrated using MAKER2 (v2.31.10; -max_dna_len 3,000,000; -min_contig 10,000; -pred_flank 500; -min_protein 30) and HiFAP (Wuhan OneMore Tech Co., Ltd., https://www.onemore-tech.com/, accessed on 9 October 2025) to generate a non-redundant reference gene set [68].

Protein-coding genes were functionally annotated based on sequence similarity searches using DIAMOND (v2.1.11.165; --evalue 1e-5 --max-hsps 1) against Swiss-Prot, TrEMBL, NR, KEGG, and KOG/COG databases [69,70,71,72,73,74]. Conserved domains and motifs were assigned with InterProScan (v5.74-105.0; --seqtype p --formats TSV --goterms --pathways -dp) and HMMER (v3.3.1; hmmsearch -E 1e-5 --domE 1e-5) against Pfam (v38.0) [75]. GO terms and KEGG pathway assignments were obtained using KOBAS (v3.0; -t blastout:tab -s ko) based on sequence similarity results [72,76,77]. Non-coding RNAs were annotated using different strategies according to their sequence and structural characteristics: tRNAs were identified with tRNAscan-SE (v1.3.1; -q), rRNAs were detected with barrnap (v0.9; --quiet --kingdom), miRNAs and snRNAs were predicted with Infernal (v1.1.5; cmscan; --rfam --nohmmonly) against Rfam (v14.8) covariance models [78,79,80,81].

### 3.4. Phylogenomic Analysis and Divergence Time Estimation

Orthology inference was performed by comparing the *P. largha* protein sequences with same species from 19 reference genomes: ten pinnipeds (*P. vitulina*, *Halichoerus grypus*, *Zalophus californianus*, *Odobenus rosmarus divergens*, *Leptonychotes weddellii*, *M. angustirostris*, *Mirounga leonina*, *N. schauinslandi* and *Eumetopias jubatus*, *Callorhinus ursinus*), eight terrestrial carnivores (*Ursus arctos*, *Ursus maritimus*, *Ursus americanus*, *Ailuropoda melanoleuca*, *Enhydra lutris kenyoni*, *Lontra canadensis*, *Lutra lutra* and *Meles meles*) and the outgroup (*Canis lupus baileyi*) (Table S7). All genome assembly and annotation files were obtained from the NCBI genome database.

Orthogroups were inferred with OrthoFinder (v2.5.5; -S blast -I 1.5) [82]. Species-specific genes were defined as sequences not assigned to any orthogroup. Single-copy orthologs shared by all species were aligned at the nucleotide level and used to infer a maximum-likelihood (ML) phylogeny with RAxML (v8.2.12; -f a -N 100 -m GTRGAMMA) [83]. Divergence times were estimated with PAML (v4.9; -clock 3, -model 0) using the ML topology and calibration points from TimeTree (v5.0) for the following pairs: *C. l. baileyi*–*U. americanus*; *L. lutra*–*L. canadensis*; *O. r. divergens*–*L. weddellii*; *A. melanoleuca*–*U. maritimus*; *O. r. divergens*–*Z. californianus*; and *H. grypus*–*M. angustirostris* [84,85].

### 3.5. Collinearity Analysis and Expansion and Contraction of Gene Families

Pairwise searches were performed with BLASTP (v2.16.0+; -evalue 1e-5) between *P. largha* and two pinniped genomes (*N. schauinslandi* and *Z. californianus*) to identify homologous gene pairs [86]. Genome-wide collinearity blocks were detected using WGDI (v0.5.6; -score 100 -evalue 1e-5 -ks_model NG86) [87]. Self-synteny analysis (*P. largha* vs. *P. largha*) was used to evaluate assembly integrity, whereas interspecies comparisons (*P. largha* vs. *N. schauinslandi*; *P. largha* vs. *Z. californianus*) were used to assess chromosomal rearrangements and macrosyntenic conservation. Syntenic relationships were visualized using JCVI (v1.1.22; --minspan 30) [88].

Gene family size evolution was examined using CAFE (v5.0.0; -P 0.05 -E 2 -I 300 -R 1) under a stochastic birth–death model of gene gain and loss [89]. *p* values were computed for each family, and significance was defined as FDR-adjusted *p* < 0.05. GO and KEGG enrichment analyses of significantly expanded or contracted families were performed in *P. largha* (FDR-adjusted *p* < 0.01).

### 3.6. Positive Selection of Genes and Identification of Gene Families

Positive selection was tested using the branch-site model (Model A) implemented in Codeml within the PAML package (v4.9; -model 2 -NSsites 2 -fix_omega 1 -omega 1.0) [90]. Single-copy orthologues identified by OrthoFinder were selected for downstream analyses to minimise confounding effects from paralogous sequences. For each orthogroup, amino-acid sequences were aligned using MAFFT (v7.505) with the L-INS-i strategy [90]. Codon alignments were subsequently generated by back-translating the protein alignments to their corresponding CDS sequences using PAL2NAL (v14) [91]. To reduce false-positive signals caused by alignment artefacts, codon alignments were subjected to stringent quality control. Poorly aligned regions were trimmed using trimAl (v2.0) [92]. Alignment columns containing more than 50% gaps were removed. Sequences containing internal stop codons or apparent frameshifts were excluded. Alignments shorter than 150 codons after trimming were discarded from further analyses. *P. largha* was designated as the foreground lineage, while all remaining species were treated as background branches. Likelihood-ratio tests (LRTs) comparing the alternative model (allowing ω > 1 on the foreground branch) with the corresponding null model (fix_omega = 1) were evaluated against a χ^2^ distribution with one degree of freedom (df = 1). Resulting *p* values were corrected for multiple testing using the false discovery rate (FDR), and genes with FDR-adjusted *p* < 0.01 were defined as positively selected genes (PSGs). For genes identified as significant under the branch-site model, positively selected codon sites were inferred using the Bayes empirical Bayes (BEB) method, and sites with posterior probability > 0.95 were regarded as candidates under positive selection. PSGs were subsequently subjected to GO and KEGG enrichment analyses (FDR-adjusted *p* < 0.01).

Given the strong enrichment of fur- and skin-adaptation-related GO terms among significantly expanded gene families and PSGs in *P. largha*, two representative families, including keratin (*KRT*) and matrix metalloproteinase (*MMP*), were selected for targeted comparative analysis in *P. largha* and the same 18 carnivoran reference species.

Family members were identified using a combined homology- and domain-based strategy. Well-annotated *KRT* and *MMP* protein sequences from *C. lupus familiaris*, *Homo sapiens* and *Mus musculus* were downloaded from the NCBI nucleotide database (https://www.ncbi.nlm.nih.gov/nuccore, accessed on 1 April 2025), and used as seed queries in BLASTP searches against the protein sets of *P. largha* and other species (*P. vitulina*, *H. grypus*, *Z. californianus*, *O. rosmarus divergens*, *L. weddellii*, *M. angustirostris*, *M. leonina*, *N. schauinslandi*, *E. jubatus*, *C. ursinus*, *U. arctos*, *U. maritimus*, *U. americanus*, *A. melanoleuca*, *E. lutris kenyoni*, *L. canadensis*, *L. lutra*, *M. meles* and *C. lupus baileyi*) (Table S7). In parallel, all predicted proteins were scanned with HMMER (v3.3.1; --cut_tc) using Pfam-A profiles (PF00038 for *KRT*; PF00413 and PF00045 for *MMP*). Candidate genes recovered by either BLAST-or HMME R-based search were merged, and sequences containing complete conserved domains, as verified by InterProScan, were retained as high-confidence family members.

For each family, the theoretical isoelectric point (pI) and molecular weight (MW) of the encoded proteins were calculated using the R package Peptides (v2.4.6). *KRT*s were classified into type I (pI < 6.1) and type II (pI ≥ 6.1). In the *MMP* family, sequences with a peptidase M10 domain were defined as matrix metallopeptidases and further divided into “WithHemopexin” or “WithoutHemopexin” according to the presence of a C-terminal hemopexin-like domain, whereas proteins lacking the M10 domain were grouped as “Others”.

### 3.7. Phylogenetic, Structural and Genomic Analyses

Multiple sequence alignments for each family were generated using MUSCLE (v3.8.1551), and maximum-likelihood phylogenies were reconstructed with IQ-TREE (v3.0.1; -m MFP -bb 1000), with the best-fitting substitution models selected by its built-in ModelFinder [93,94]. Conserved motifs were identified using MEME Suite (v5.5.9; -nmotifs 10 -minw 6 -maxw 200), and protein domains were annotated using NCBI Batch CD-Search against the Conserved Domain Database (https://www.ncbi.nlm.nih.gov/Structure/bwrpsb/bwrpsb.cgi/, accessed on 9 October 2025) [95].

To characterise genomic organisation, genomic coordinates of all *KRT*s and *MMP*s in *P. largha* were extracted from the genome annotation file, and gene locations were visualised using the TBtools (v2.323) and R package RIdeogram (v0.2.2) [96,97]. Intraspecies collinearity was examined using MCScanX (v1.0.0) to detect potential tandem or segmental duplication events involving *KRT* and *MMP* gene families within the *P. largha* genome [98]. For interspecies comparisons, orthologous gene pairs and conserved collinear blocks were identified between *P. largha* and six representative species (*C. lupus baileyi*, *A. melanoleuca*, *L. lutra*, *M. meles*, *N. schauinslandi* and *Z. californianus*). The resulting synteny maps were used to evaluate structural conservation and lineage-specific rearrangements of the *KRT* and *MMP* gene families.

## 4. Conclusions

In this study, a near-telomere-to-telomere (near-T2T), gap-free reference genome was generated for the spotted seal (*Phoca largha*), providing a chromosome-complete framework for genomic analyses in this ice-associated pinniped. Using comparative genomics across pinnipeds and terrestrial carnivores, lineage-specific gene-family changes and positively selected genes were identified in *P. largha*, with functional signals repeatedly enriched for integumentary and extracellular matrix processes associated with skin and fur biology.

Building on these genome-wide patterns, targeted analyses of two representative families, keratins (*KRT*) and matrix metalloproteinases (*MMP*), revealed distinct evolutionary and genomic organisation features: *KRT* genes were concentrated in major chromosomal clusters and exhibited conserved locus-level synteny with lineage-dependent remodelling within clusters, whereas *MMP* genes showed a dispersed chromosomal distribution and broadly conserved orthologous correspondence across species. Together, these results suggest that variation of potential adaptive relevance in *P. largha* may involve coordinated changes at multiple genomic levels, including gene-family turnover and sequence-level modification, particularly in pathways linked to epidermal structure and tissue remodelling.

This high-quality genome presented here establishes an essential resource for future studies of pinniped genome evolution, structural variation and the genetic basis of cold and aquatic adaptation and will facilitate conservation and population genomic investigations of *P. largha*.

## Figures and Tables

**Table 1:** Statistics and quality evaluation of the *P. largha* genome.

	Initial Assembly	Contig Anchoring	Final Assembly
Assembly level	contig	chromosome	complete
Total length (bp)	2,399,564,555	2,412,794,123	2,394,157,917
No. of chromosome	-	16	16
No. of contig	69	40	16
Contig N50 (bp)	84,603,143	115,281,119	184,385,724
Scaffold N50 (bp)	84,603,143	157,209,874	184,385,724
Mapping rate			
DNBSEQ short reads	99.99% (99.98% coverage)		
PacBio long reads	100.00% (99.96% coverage)		
ONT long reads	99.95% (99.60% coverage)		
Merqury QV score			
DNBSEQ short reads	53.77		
PacBio long reads	55.82		
BUSCO evaluation	13,727 (carnivora_odb12)		
Complete BUSCOs	99.34%		

**Table 2:** Statistics of chromosomes and telomeres in the *P. largha* genome.

Chromosome	Length (bp)	No. of Contig	No. of Gaps	No. of Start Repeat Unit	No. of End Repeat Unit
chr1	111,367,719	1	0	1457	2262
chr2	61,262,870	1	0	1473	1441
chr3	139,520,123	1	0	1344	1454
chr4	147,021,747	1	0	0	1132
chr5	93,748,336	1	0	2907	1582
chr6	94,184,855	1	0	539	253
chr7	108,831,544	1	0	1475	4
chr8	205,021,079	1	0	2209	1991
chr9	217,726,678	1	0	1046	2594
chr10	211,422,629	1	0	0	1694
chr11	195,801,467	1	0	1981	1481
chr12	190,005,403	1	0	2091	1092
chr13	184,385,724	1	0	1927	665
chr14	145,726,044	1	0	2453	1422
chr15	157,258,176	1	0	1284	2463
chr16	130,873,523	1	0	2663	0

## Data Availability

All raw sequencing data have been uploaded to the China National Centre for Bioinformation (CNCB) with the accession number CRA037495 (DNBSEQ raw data: CRX2482382, PacBio raw data: CRX2482383, ONT raw data: CRX2482384, Hi-C raw data: CRX2482385, RNA-seq raw data: CRX2482386) [99,100,101]. The final chromosome-level assembly and annotation files have been uploaded to the figshare database (https://doi.org/10.6084/m9.figshare.31132450, accessed on 23 January 2026) [101]. No specific code was used in this study. The data analyses adhered to the manuals and protocols offered by the creators of the corresponding bioinformatics tools, the parameter settings of which were outlined in the Section 3.

## Supplementary Materials

The following supporting information can be downloaded at: https://www.mdpi.com/article/10.3390/ijms27062618/s1.
