# Supplementary material for: A Near-Telomere-to-Telomere Genome Assembly of the Spotted Seal (Phoca largha) Reveals Genomic Architecture Underlying Skin and Fur Adaptation
Source: Int J Mol Sci. 2026 Mar 13;27(6):2618. doi: 10.3390/ijms27062618 (PMC13026343; doi:10.3390/ijms27062618)
Supplement: Supplementary file 1 [file ijms-27-02618-s001.zip › Figure S4.pdf]

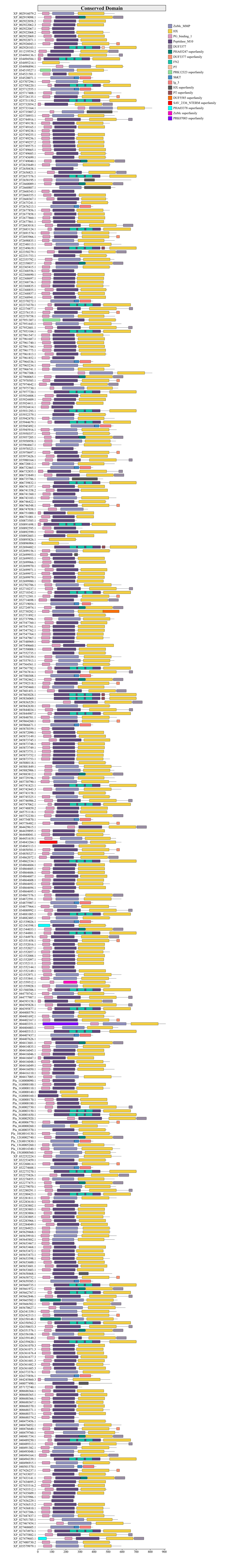
Conserved Domain
ZnMc_MMP
HX
PG_binding_1
Peptidase_M10
DUF3377
PHA03247 superfamily
DUF3377 superfamily
FN2
PT
PRK12323 superfamily
ShKT
Ig_3
HX superfamily
PT superfamily
DUF5585 superfamily
SAV_2336_NTERM superfamily
PHA03378 superfamily
ZnMc superfamily
PRK07003 superfamily
0
100
200
300
400
500
600
700
800
900
