# Supplementary material for: A Near-Telomere-to-Telomere Genome Assembly of the Spotted Seal (Phoca largha) Reveals Genomic Architecture Underlying Skin and Fur Adaptation
Source: Int J Mol Sci. 2026 Mar 13;27(6):2618. doi: 10.3390/ijms27062618 (PMC13026343; doi:10.3390/ijms27062618)
Supplement: Supplementary file 1 [file ijms-27-02618-s001.zip › ijms-4146337-supplementary.pdf]

Supplementary Materials

A Near-Telomere-to-Telomere Genome Assembly of the Spotted Seal (Phoca largha) Reveals Genomic Architecture Underlying Skin and Fur Adaptation

This file includes:

Figures S1 to S2

Tables S1 to S8

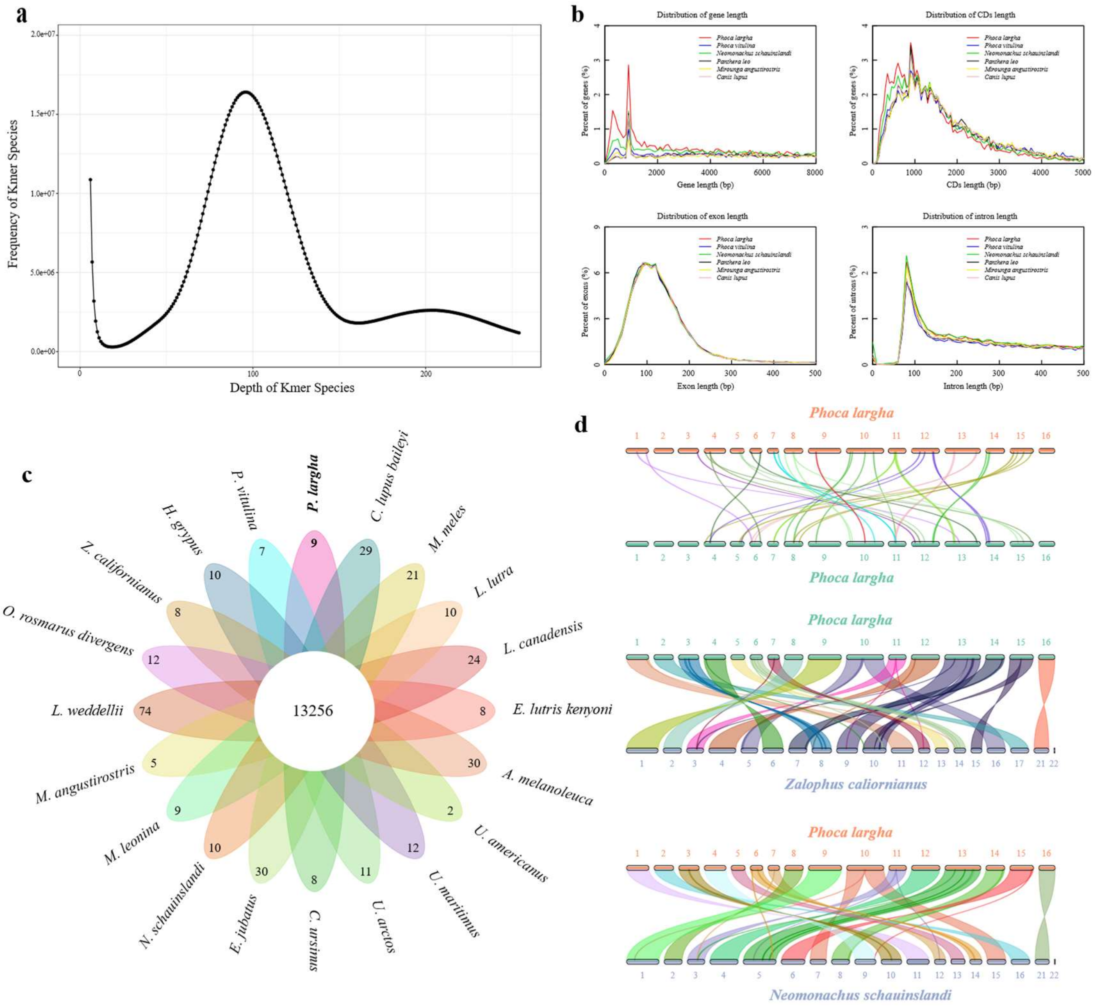

Figure S1 Gene structural features and genomic comparisons across species. (a) K-mer (17-mer) distribution and estimation of genome size in *P. largha*. (b) Comparative analysis of gene length,

CDs length, exon length and intron length across five closely related species. (c) The Venn diagram of shared and unique gene families among nine species. (e) Genome collinearity analysis among *P. largha* and two other pinniped species (*Z. californianus* and *N. schauinslandi*).

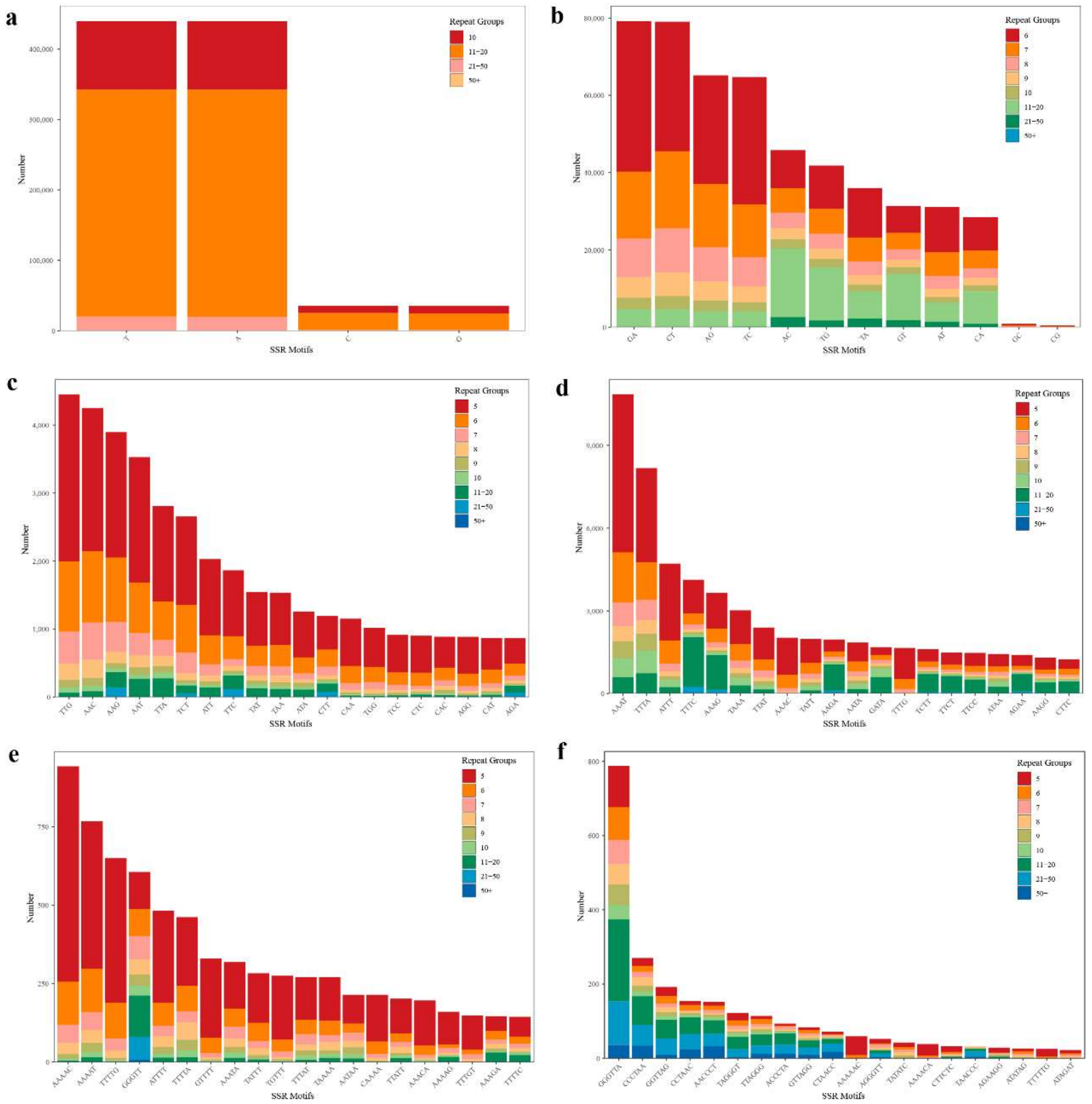

Figure S2 Distribution of repeat numbers across different simple sequence repeats (SSRs) motif types in the *P. largha* genome. (a) mononucleotide repeats. (b) dinucleotide repeats. (c) trinucleotide repeats. (d) tetranucleotide repeats. (e) pentanucleotide repeats. (f) hexanucleotide repeats. Different colours represent different repeat groups.

Table S1 Statistics of sequencing data.

Table S1a Summary of sequencing libraries.

Libraries	Clean Read Number	Clean Data (Gb)	Read Length (bp)	GC Content (%)
DNBSEQ Reads	1,847,106,362	266.78	144	40.03
PacBio Reads	5,928,559	98.71	16,649	40.15
ONT Reads	3,000,615	112.05	37,340	42.10
Hi-C Reads	1,767,939,780	262.86	150	42.05
RNA-seq Reads	25,285,483	7.59	150	48.87

Table S1b Statistics of length distribution from ONT ultra-long reads.

Length Cutoff	Total number	Total bases (bp)	Max length (bp)	Mean length (bp)	N50 (bp)	N90 (bp)	GC content (%)
all	3,000,615	112,045,333,347	724,714	37,340	67,667	26,003	42.10
≥50 kb	966,941	78,724,647,484	724,714	81,416	82,026	56,234	42.10
≥75 kb	453,460	47,114,322,742	724,714	103,899	100,923	79,168	42.20
≥100 kb	185,807	24,164,166,029	724,714	130,049	125,170	103,808	42.20

Table S2 Statistics of *P. largha* genome assemblies.

Assembly	This study	Early edition 1	early edition 2	early edition 3	early edition 4
Total Length (Gb)	2.39	2.26	2.36	2.39	2.41
No. of Chromosome	16	-	16	-	16
No. of Scaffold	16	50,053	249,399	-	752
Scaffold N50 (Mb)	184.39	87.53	142.1	59.25	179.7
Assembly Level	Near-Complete	Scaffold	Chromosome	Scaffold	Chromosome
GC Content (%)	41.54	41.36%	41.36%		
Repeats Content (%)	39.91	35.83	-	39.73	44.91
BUSCO Completeness (%)	99.34	89.0%	91.4%	-	95.00%

Table S3 Summary of centromeres in *P. largha* genome.

Chromosome	Start	End	Centromeric Length (bp)	Start_TRF	End_TRF
chr1	57,399,241	59,413,828	2,014,588	TR_00122	TR_00138
chr2	18,647,353	18,959,185	3,11,833	TR_00090	TR_00077
chr3	120,001,789	120,863,805	862,017	TR_00231	TR_00120
chr4	142,730,197	144,693,194	1,962,998	TR_00299	TR_00106
chr5	83,231,675	84,438,794	1,207,120	TR_00138	TR_00097
chr6	1	596,242	596,242	TR_00109	TR_00150
chr7	48,345,386	48,680,811	335,426	TR_00116	TR_00148
chr8	85,624,007	115,689,967	30,065,961	TR_00143	TR_00320
chr9	156,930,331	159,851,639	2,921,309	TR_00290	TR_00218
chr10	176,330,479	179,218,991	2,888,513	TR_00441	TR_00144
chr11	114,318,950	115,128,951	810,002	TR_00137	TR_00193
chr12	155,960,874	156,107,688	146,815	TR_00198	TR_00093
chr13	56,838,517	59,387,161	2,548,645	TR_00422	TR_00359
chr14	110,278,181	114,998,262	4,720,082	TR_00117	TR_00150
chr15	156,304,444	157,232,945	928,502	TR_00287	TR_00160
chr16	129,061,430	130,871,652	1,810,223	TR_00448	TR_00407

Table S4 Statistics of repetitive sequences in the *P. largha* genome.

Type	Length (bp)	Percentage of Genome (%)
DNA transposon	91,876,925	3.84
LINE	664,630,466	27.76
SINE	62,122,015	2.59
LTR	138,740,424	5.79
Satellite	2,258,114	0.09
Simple repeat	0	0.00
Other	2,479	0.00

Unknown	17,071,890	0.71
Total	955,489,465	39.91

Table S5 Statistics of SSRs in the *P. largha* genome.

Type	Number	Percentage of SSR Loci (%)
mononucleotide repeats	950,103	58.97
dinucleotide repeats	503,835	31.27
trinucleotide repeats	54,133	3.36
tetranucleotide repeats	89,094	5.53
pentanucleotide repeats	10,963	0.68
hexanucleotide repeats	3,381	0.21
total	1,611,509	100

Note: a total of 280,887 SSRs were identified as compound microsatellites.

Table S6 Statistics of the noncoding RNA in the *P. largha* genome.

Type		Copy Number	Average Length (bp)	Total Length (bp)	Percentage of Genome (%)
miRNA		821	74	61,100	0.002552
tRNA		698	74.34	51,891	0.002167
rRNA	Total	416	204	84,741	0.003539
	18S	11	1,496	16,458	0.000687
	28S	11	2,759	30,352	0.001268
	5.8S	8	147	1,176	0.000049
	5S	386	95	36,755	0.001535
snRNA	Total	2,638	114	299,565	0.012512
	CD-box	484	92	44,718	0.001868
	HACA-box	279	137	38,254	0.001598
	splicing	1,846	115	211,909	0.008851

scaRNA	29	162	4,684	0.000196

Table S7 Species information used in this study.

Species Name (Scientific Name)	Taxonomic Order	Taxonomic Family	Data source
**For homology-based prediction in genome annotation**			
Phoca vitulina (harbour seal)	Carnivora	Phocidae	https://ftp.ncbi.nlm.nih.gov/genomes/all/GCF/004/348/235/GCF_004348235.1_GSC_HSeal_1.0/
Mirounga angustirostris (northern elephant seal)	Carnivora	Phocidae	https://ftp.ncbi.nlm.nih.gov/genomes/all/GCF/029/215/605/GCF_029215605.1_mMirAng1.0.hap1/
Neomonachus schauinslandi (Hawaiian monk seal)	Carnivora	Phocidae	https://ftp.ncbi.nlm.nih.gov/genomes/all/GCF/002/201/575/GCF_002201575.2_ASM220157v2/
Panthera leo (Lion)	Carnivora	Felidae	https://ftp.ncbi.nlm.nih.gov/genomes/all/GCF/018/350/215/GCF_018350215.1_P.leo_Ple1_pat1.1/
Canis lupus familiaris (dog)	Carnivora	Canidae	https://ftp.ncbi.nlm.nih.gov/genomes/all/GCF/011/100/685/GCF_011100685.1_UU_Cfam_GSD_1.0/
**For phylogenetic analysis in comparative genomic analyses and gene family analyses**			
Phoca vitulina (harbour seal)	Carnivora	Phocidae	https://ftp.ncbi.nlm.nih.gov/genomes/all/GCF/004/348/235/GCF_004348235.1_GSC_HSeal_1.0/
Halichoerus grypus (grey seal)	Carnivora	Phocidae	https://ftp.ncbi.nlm.nih.gov/genomes/all/GCF/012/393/455/GCF_012393455.1_Tufts_HGry_1.1/
Zalophus californianus	Carnivora	Otariidae	https://ftp.ncbi.nlm.nih.gov/genomes/all/

(California sea lion)			GCF/009/762/305/GCF_009762305.2_ mZalCal1.pri.v2/
Odobenus rosmarus divergens (Pacific walrus)	Carnivora	Odobenidae	https://ftp.ncbi.nlm.nih.gov/genomes/all/ GCF/000/321/225/GCF_000321225.1_ Oros_1.0/
Leptonychotes weddellii (Weddell seal)	Carnivora	Phocidae	https://ftp.ncbi.nlm.nih.gov/genomes/all/ GCF/000/349/705/GCF_000349705.1_L epWed1.0/
Mirounga angustirostris (northern elephant seal)	Carnivora	Phocidae	https://ftp.ncbi.nlm.nih.gov/genomes/all/ GCF/029/215/605/GCF_029215605.1_ mMirAng1.0.hap1/
Mirounga leonina (southern elephant seal)	Carnivora	Phocidae	https://ftp.ncbi.nlm.nih.gov/genomes/all/ GCF/011/800/145/GCF_011800145.1_K U_Mleo_1.0/
Neomonachus schauinslandi (Hawaiian monk seal)	Carnivora	Phocidae	https://ftp.ncbi.nlm.nih.gov/genomes/all/ GCF/002/201/575/GCF_002201575.2_ ASM220157v2/
Eumetopias jubatus (Steller sea lion)	Carnivora	Otariidae	https://ftp.ncbi.nlm.nih.gov/genomes/all/ GCF/004/028/035/GCF_004028035.1_ ASM402803v1/
Callorhinus ursinus (northern fur seal)	Carnivora	Otariidae	https://ftp.ncbi.nlm.nih.gov/genomes/all/ GCF/003/265/705/GCF_003265705.2_ GSC_fseal_2.0/
Ursus arctos (brown bear)	Carnivora	Ursidae	https://ftp.ncbi.nlm.nih.gov/genomes/all/ GCF/023/065/955/GCF_023065955.2_ UrsArc2.0/
Ursus maritimus (polar bear)	Carnivora	Ursidae	https://ftp.ncbi.nlm.nih.gov/genomes/all/ GCF/017/311/325/GCF_017311325.1_A SM1731132v1/

Ursus americanus (American black bear)	Carnivora	Ursidae	https://ftp.ncbi.nlm.nih.gov/genomes/all/GCF/020/975/775/GCF_020975775.1_gsc_jax_bbear_1.0/
Ailuropoda melanoleuca (giant panda)	Carnivora	Ursidae	https://ftp.ncbi.nlm.nih.gov/genomes/all/GCF/002/007/445/GCF_002007445.2_ASM200744v3/
Enhydra lutris kenyoni (sea otter)	Carnivora	Mustelidae	https://ftp.ncbi.nlm.nih.gov/genomes/all/GCF/002/288/905/GCF_002288905.1_ASM228890v2/
Lontra canadensis (North American river otter)	Carnivora	Mustelidae	https://ftp.ncbi.nlm.nih.gov/genomes/all/GCF/010/015/895/GCF_010015895.1_GSC_riverotter_1.0/
Lutra lutra (Eurasian otter)	Carnivora	Mustelidae	https://ftp.ncbi.nlm.nih.gov/genomes/all/GCF/902/655/055/GCF_902655055.1_mLutLut1.2/
Meles meles (European badger)	Carnivora	Mustelidae	https://ftp.ncbi.nlm.nih.gov/genomes/all/GCF/922/984/935/GCF_922984935.1_mMelMel3.1_paternal_haplotype/
Canis lupus baileyi (Mexican wolf)	Carnivora	Canidae	https://ftp.ncbi.nlm.nih.gov/genomes/all/GCF/048/164/855/GCF_048164855.1_mCanLup2.hap1/
**For blast search in gene family analyses**			
Canis lupus familiaris (dog)	Carnivora	Canidae	https://www.ncbi.nlm.nih.gov/nuccore/
Homo sapiens (human)	Primates	Hominidae	https://www.ncbi.nlm.nih.gov/nuccore/
Mus musculus (home mouse)	Rodentia	Muridae	https://www.ncbi.nlm.nih.gov/nuccore/

Table S8 Statistics of results of genome collinearity analysis.

Species vs Species	Syntenic Blocks	Average Syntenic Gene Pairs Per Block	Gene Pairs Included in Syntenic Block	Median Block Length (bp)
P. largha vs *P. largha*	394	8	3,070	1,170,280/1,183,443
P. largha vs *Z. californianus*	325	61	19,721	626,906/732,956
P. largha vs *N. schauinslandi*	301	65	19,506	649,260/826,599

Other Figture legends

Figture S3 Comparison of KRT gene domains across *P. largha* and 19 other species.

Figture S4 Comparison of MMP gene domains across *P. largha* and 19 other species.

Other Table legends

Table S9 Significantly enriched GO terms of the expanded gene families.

Table S10 Significantly enriched KEGG pathways of the expanded gene families.

Table S11 Positively selected genes of the spotted seal compared with the other species.

Table S12 Significantly enriched ($P<0.01$) GO terms of the positively selected genes of the spotted seal.

Table S13 Significantly enriched ($P<0.01$) KEGG terms of the positively selected genes of the spotted seal.

Table S14 Summary of KRT and MMP gene family information across *P. largha* and 19 other species. (i) Classification and identification of the KRT gene family, with the total number of families and their distribution across categories. (ii) Classification and identification of the MMP gene family, with the total number of families and their distribution across categories. (iii) Details for each KRT gene family member: gene ID, chromosome location, start and end positions, amino acid length, molecular weight, and isoelectric point. (iv) Details for each MMP gene family member: gene ID, chromosome location, start and end positions, amino acid length, molecular weight, and isoelectric point.
