# Supplementary figures and images for: A Near-Telomere-to-Telomere Genome Assembly of the Spotted Seal (Phoca largha) Reveals Genomic Architecture Underlying Skin and Fur Adaptation
Source: Int J Mol Sci. 2026 Mar 13;27(6):2618. doi: 10.3390/ijms27062618 (PMC13026343; doi:10.3390/ijms27062618)

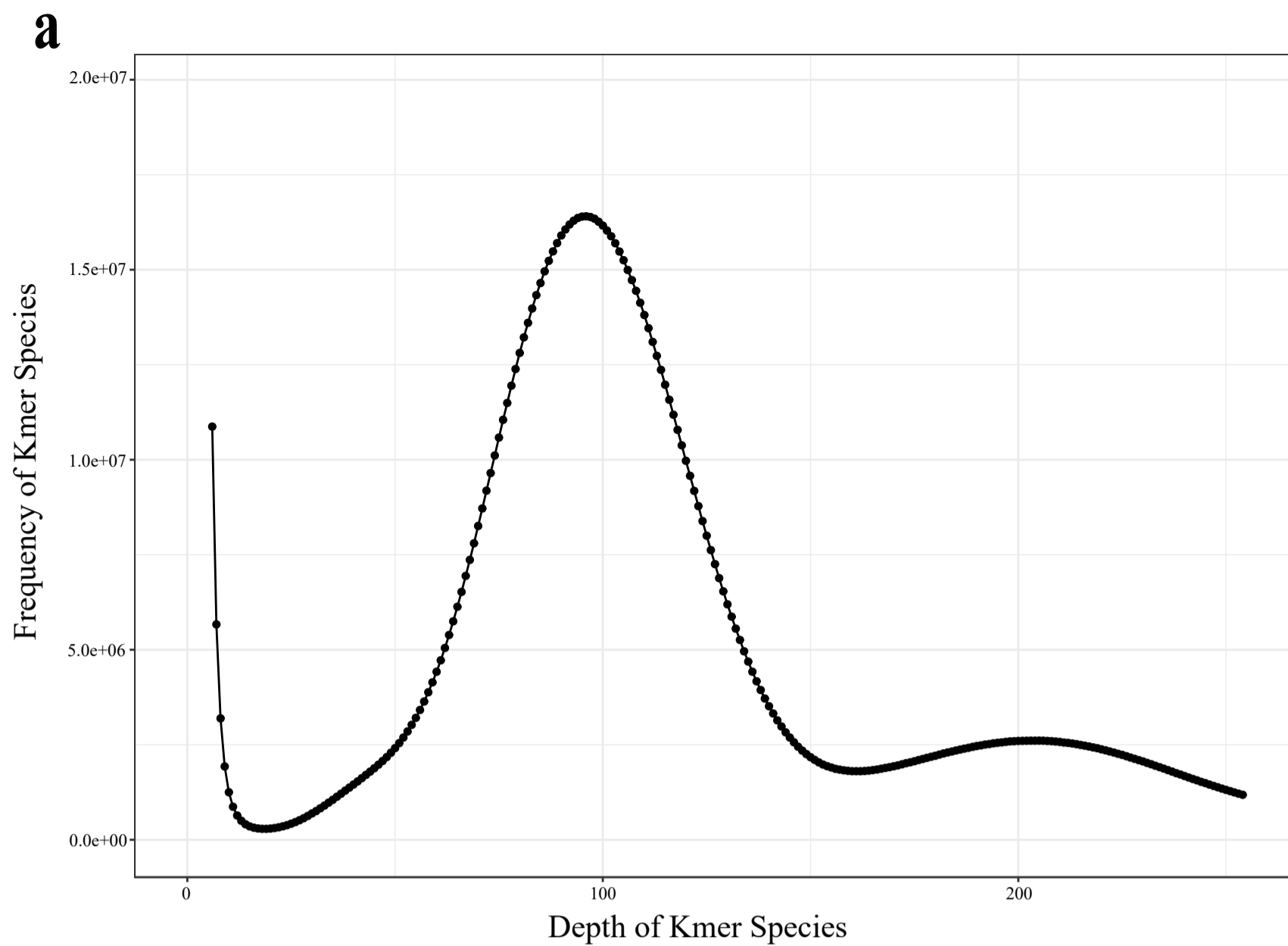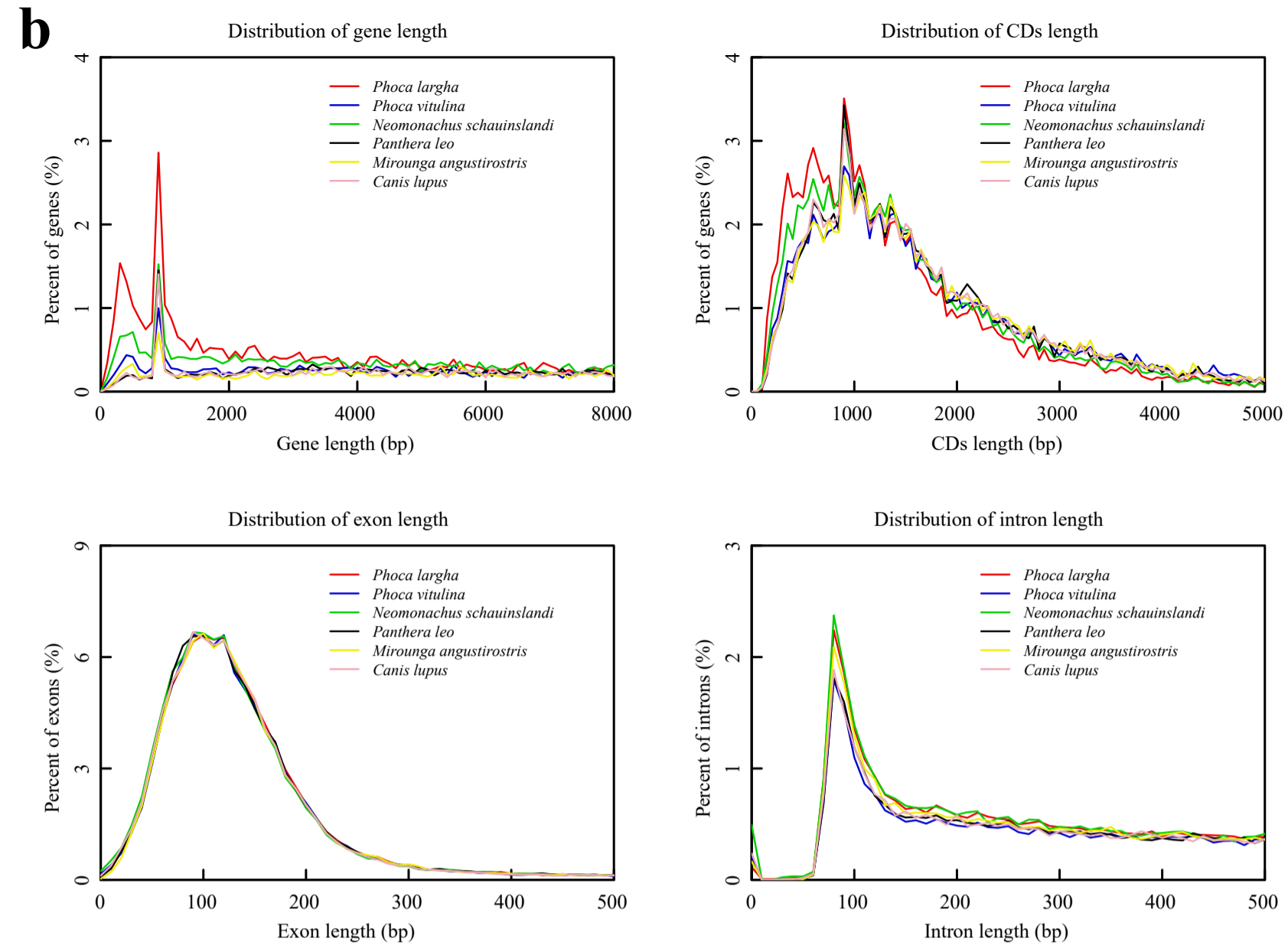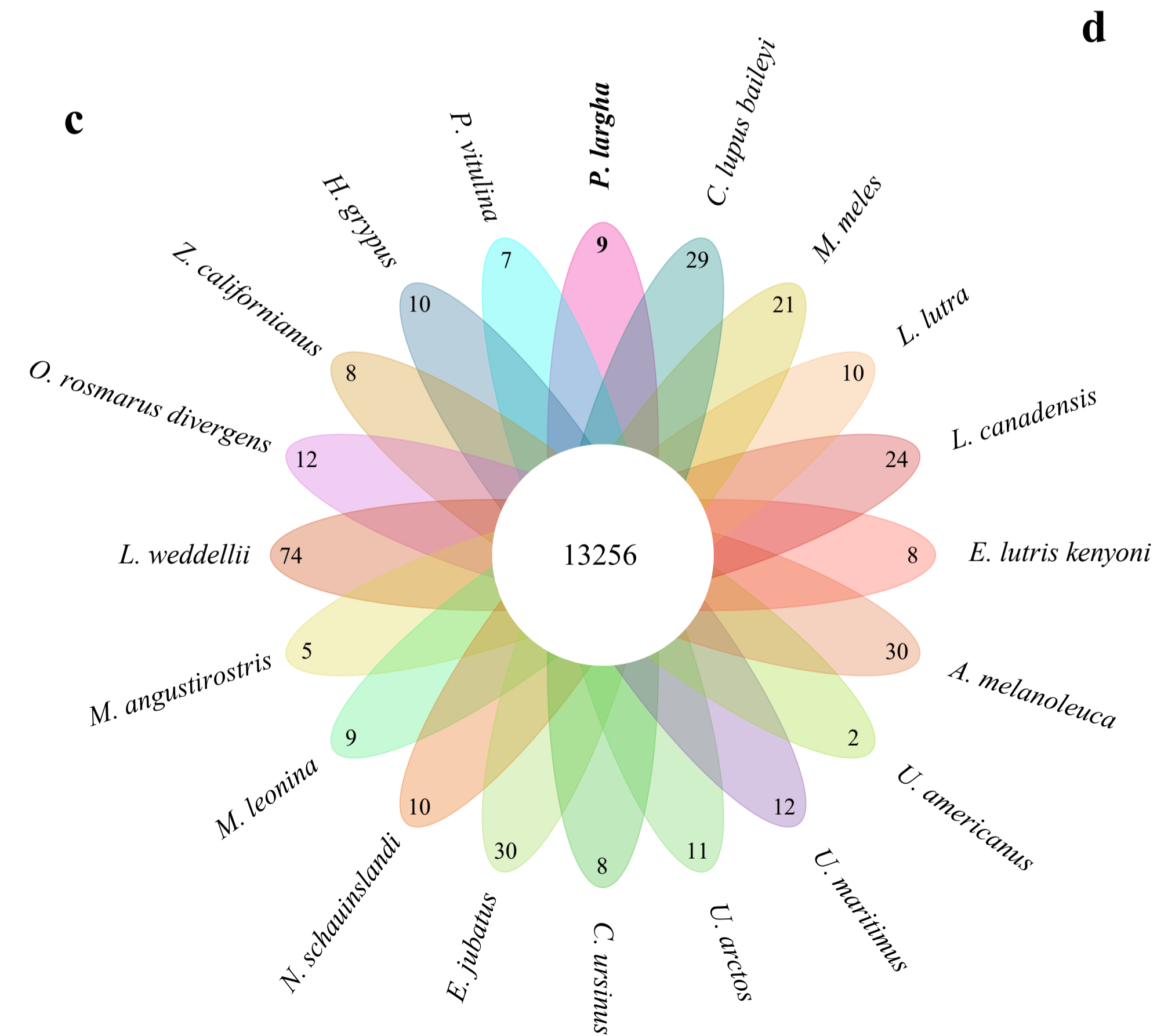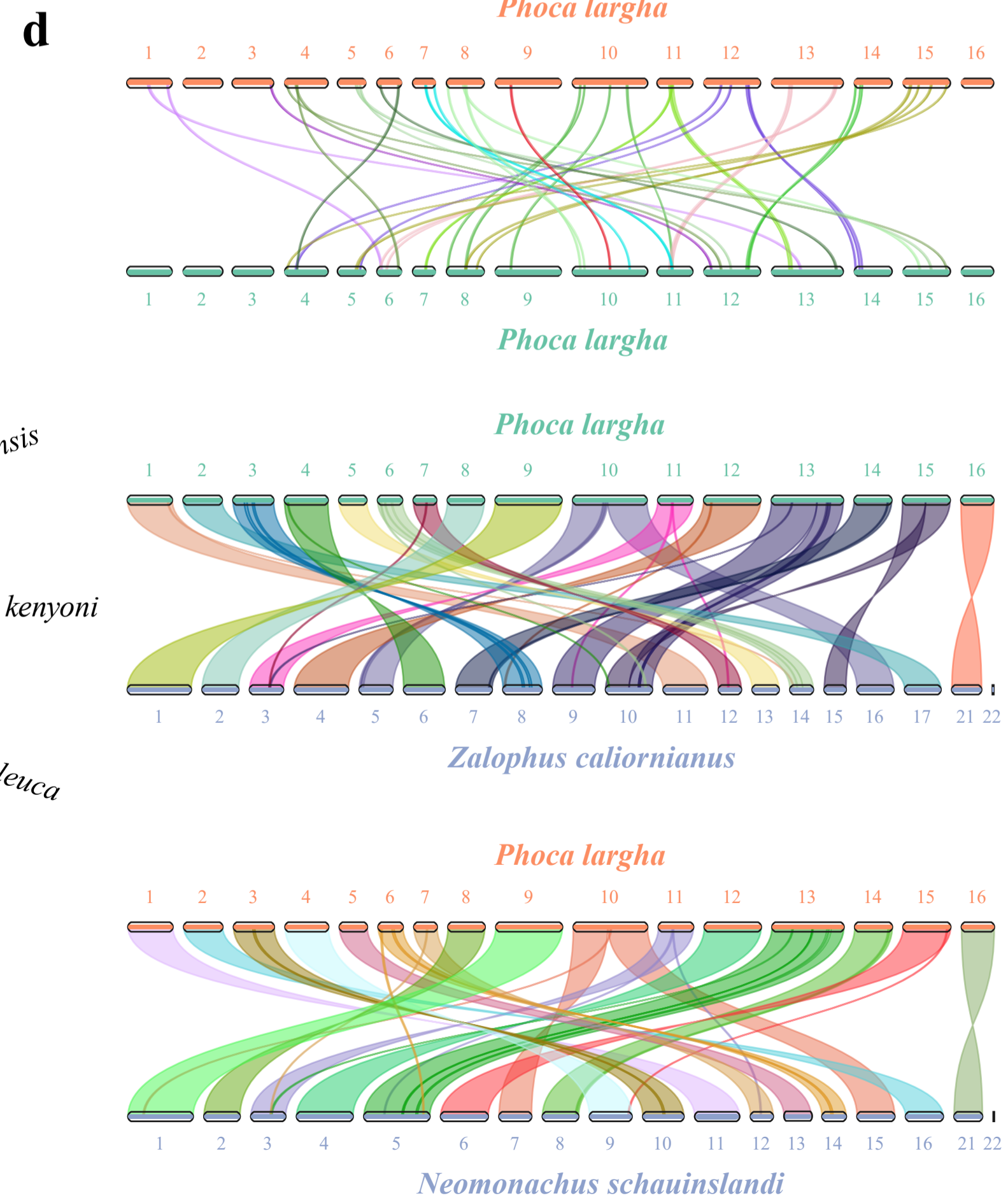

Supplement: Supplementary file 1 [file ijms-27-02618-s001.zip › Figure S1.pdf]

**a**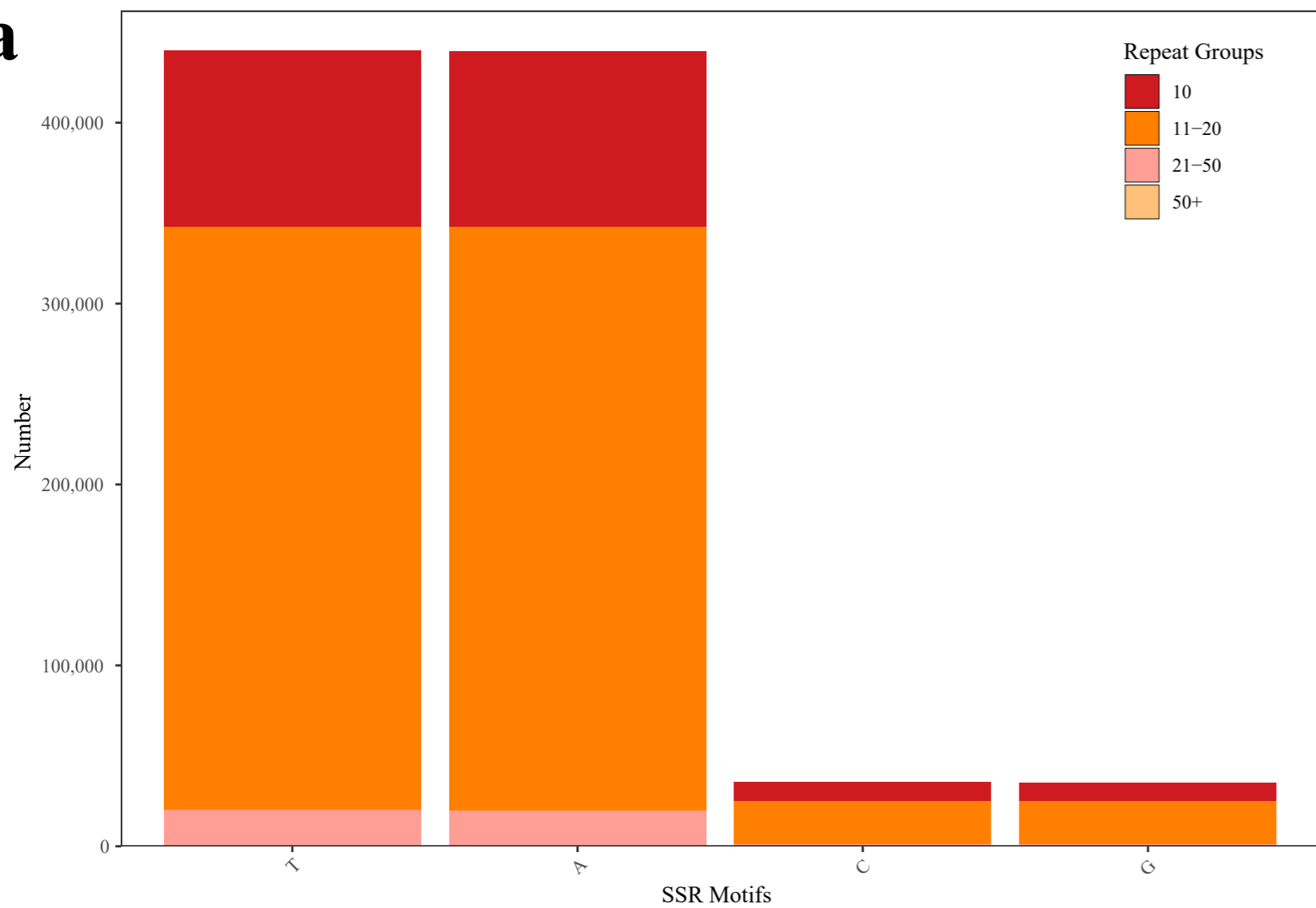**b**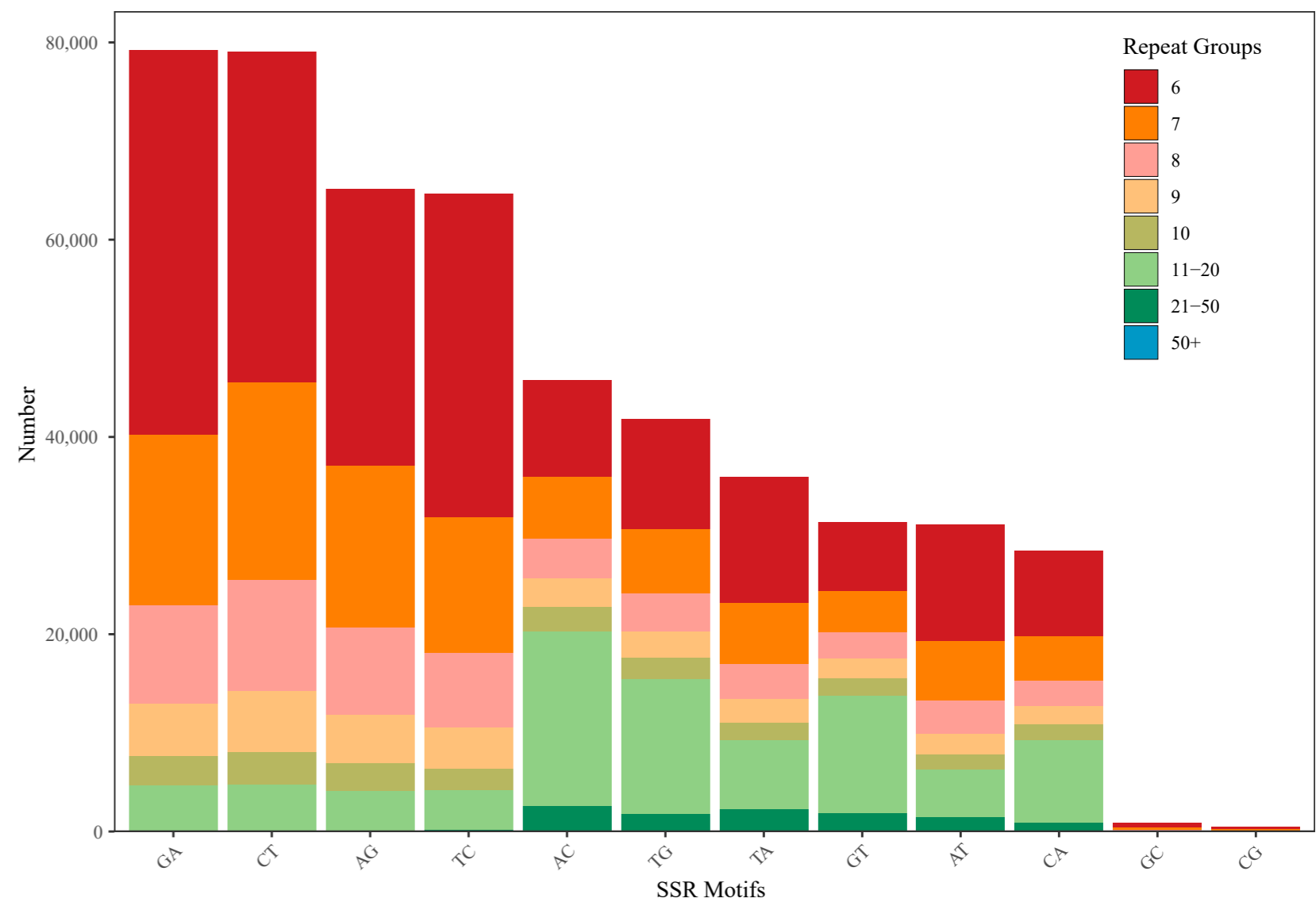**c**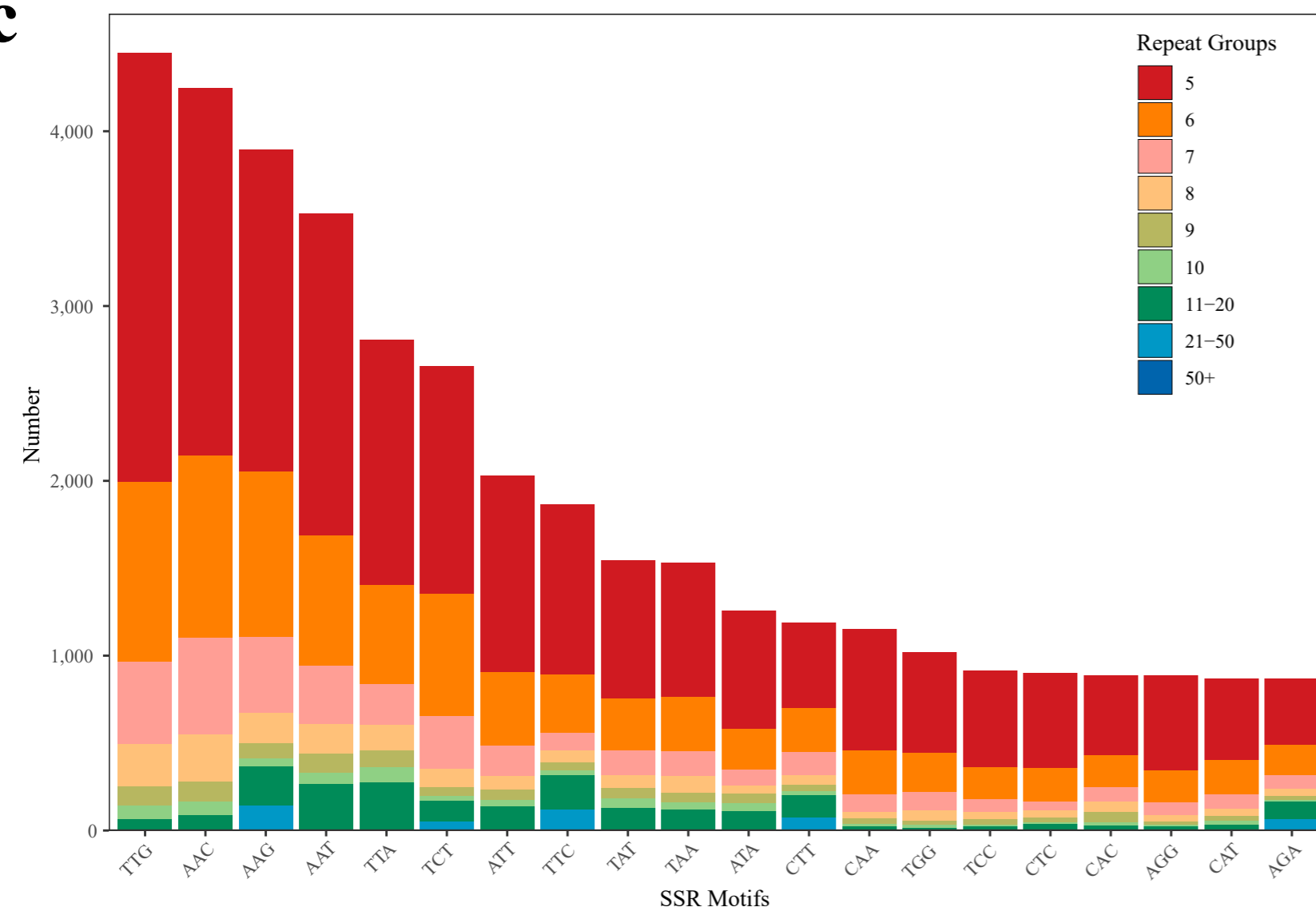**d**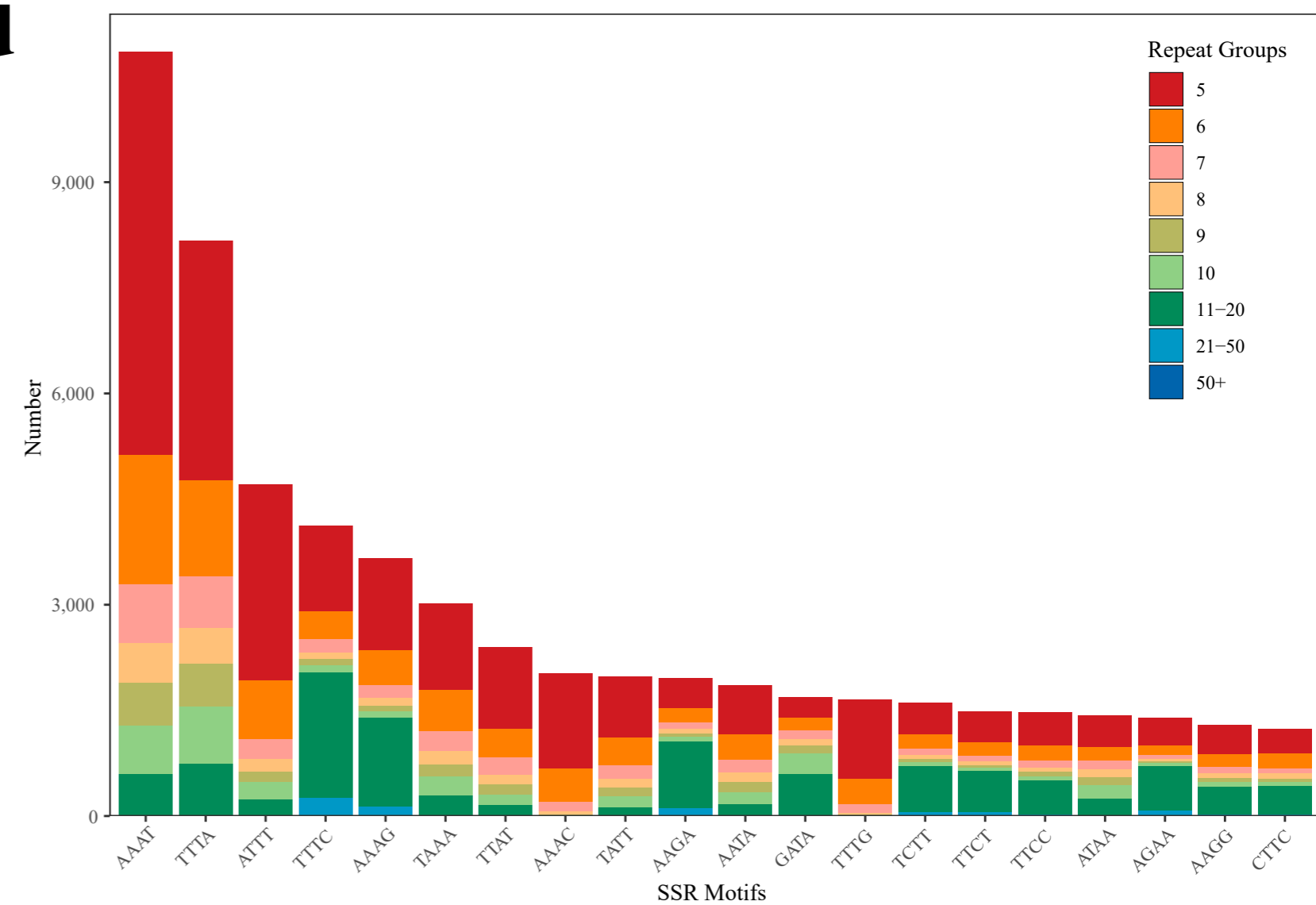**e**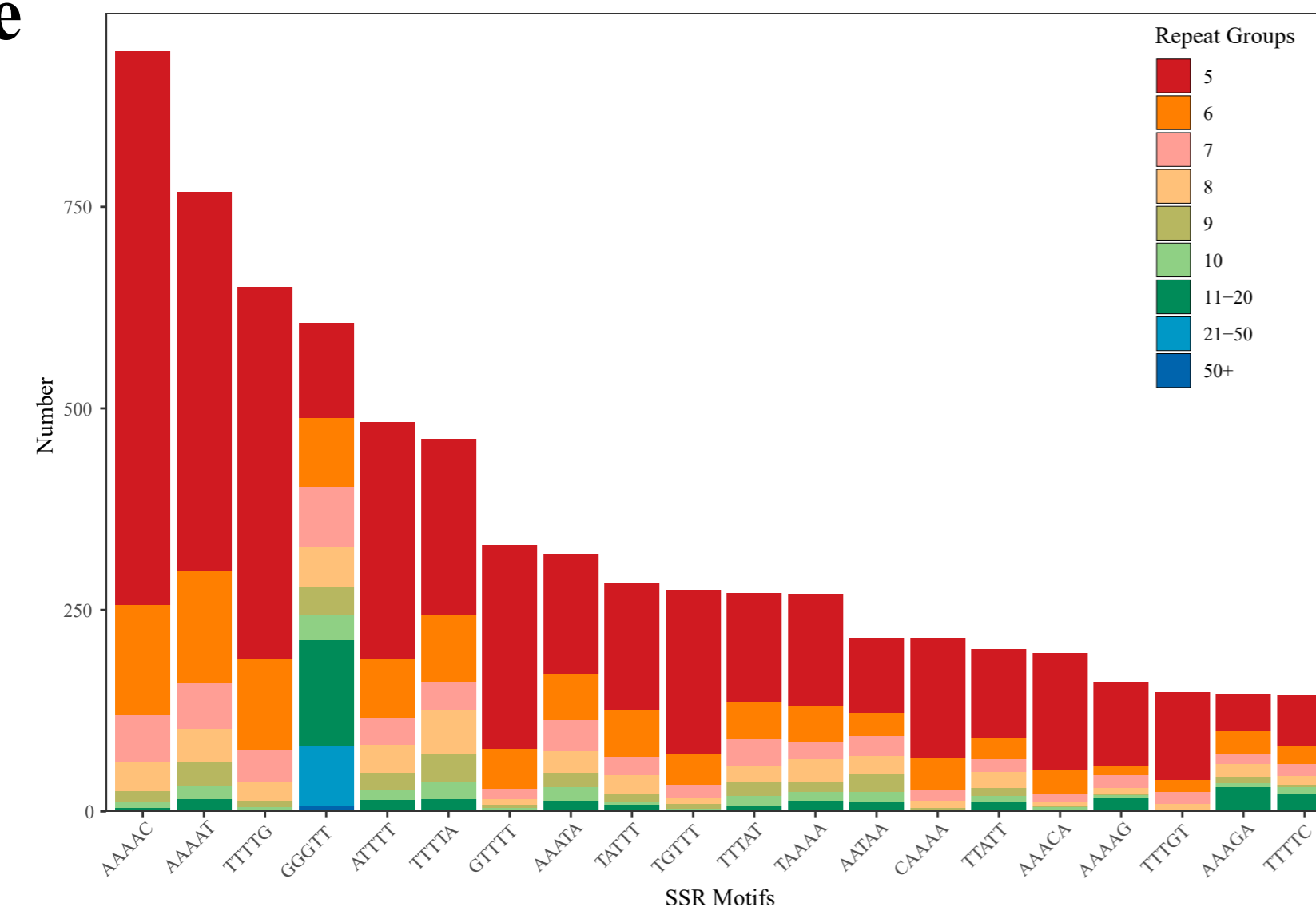**f**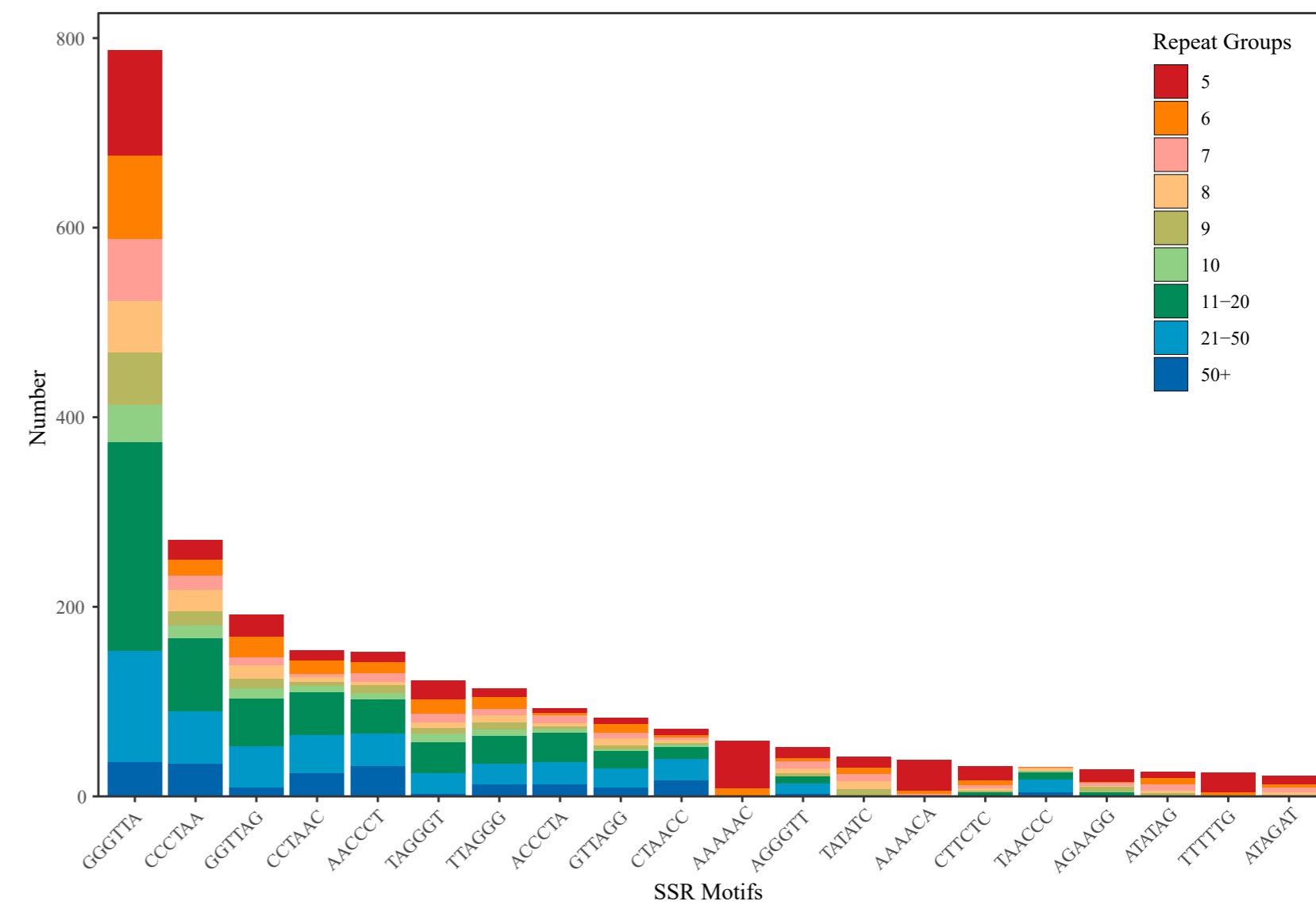

Supplement: Supplementary file 1 [file ijms-27-02618-s001.zip › Figure S2.pdf]
