# Supplementary material for: A Near-Telomere-to-Telomere Genome Assembly of the Spotted Seal (Phoca largha) Reveals Genomic Architecture Underlying Skin and Fur Adaptation
Source: Int J Mol Sci. 2026 Mar 13;27(6):2618. doi: 10.3390/ijms27062618 (PMC13026343; doi:10.3390/ijms27062618)
Supplement: Supplementary file 1 [file Figure S3.pdf › Figure S3.pdf]

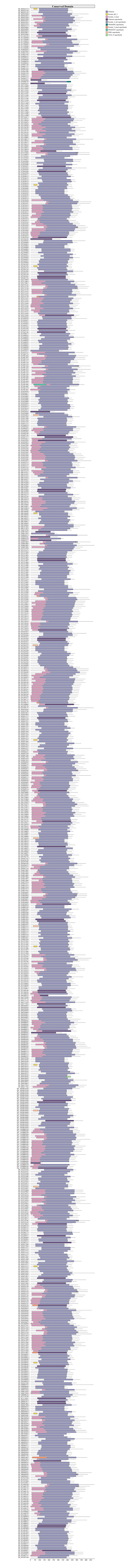

Conserved Domain
Filament
Keratin_B2_2
Keratin_2_head
Filament superfamily
Keratin_2_tail superfamily
PHA02682 superfamily
Keratin_2_head superfamily
PHA03247 superfamily
PMG superfamily
TAXi_N superfamily
XP_002922157.2
XP_002922158.2
XP_002922159.2
XP_002922206.2
XP_002923287.2
XP_002923289.2
XP_002923290.2
XP_002923291.2
XP_002923292.1
XP_002923293.2
XP_002923317.2
XP_002924988.1
XP_002924990.1
XP_002924991.2
XP_002925007.1
XP_002925008.2
XP_011226156.2
XP_011226950.2
XP_011228200.2
XP_011228203.2
XP_011228204.2
XP_011228205.2
XP_011228206.2
XP_011228208.2
XP_019658262.2
XP_019659362.2
XP_019661193.2
XP_019661194.1
XP_034495612.1
XP_034495623.1
XP_034496669.1
XP_034496670.1
XP_034496942.1
XP_034497130.1
XP_034497131.1
XP_034497141.1
XP_034497206.1
XP_034501208.1
XP_034501210.1
XP_034501212.1
XP_034501213.1
XP_034501677.1
XP_034502448.1
XP_034504574.1
XP_034507635.1
XP_025715056.1
XP_025715058.1
XP_025715059.1
XP_025715105.1
XP_025715106.1
XP_025715107.1
XP_025715108.1
XP_025715197.1
XP_025715198.1
XP_025715199.1
XP_025717535.2
XP_025717590.2
XP_025717643.1
XP_025717644.1
XP_025717659.1
XP_025717662.1
XP_025717696.2
XP_025717698.2
XP_025717699.2
XP_025738417.1
XP_025738419.1
XP_025738420.1
